# SUMOylation of PDPK1 Is required to maintain glycolysis-dependent CD4 T-cell homeostasis

**DOI:** 10.1038/s41419-022-04622-1

**Published:** 2022-02-24

**Authors:** Fei Sun, Fa-Xi Wang, He Zhu, Tian-Tian Yue, Chun-Liang Yang, Jia-Hui Luo, Xi Luo, Hai-Feng Zhou, Shan-Jie Rong, Wan-Ying Lu, Qing Zhou, Ping Yang, Fei Xiong, Yan-Jun Liu, Tong Yan, Yun-Fei Liao, Shu Zhang, Cong-Yi Wang

**Affiliations:** 1 The Center for Biomedical Research, Department of Respiratory and Critical Care Medicine, Key Laboratory of Pulmonary Diseases of Health Ministry, Tongji Hospital, Tongji Medical College, Huazhong University of Sciences and Technology, Wuhan, China; 2 The Center for Obesity and Metabolic Health, Affiliated Hospital of Southwest Jiaotong University, the Third People’s Hospital of Chengdu, 82 Qinglong Road, Chengdu, Sichuan China; 3 The Center of Gastrointestinal and Minimally Invasive Surgery, Department of General Surgery, The Third People’s Hospital of Chengdu & The Affiliated Hospital of Southwest Jiaotong University, Chengdu, Sichuan China; 4 Department of Endocrinology, Wuhan Union Hospital, Tongji Medical College, Huazhong University of Science and Technology, Wuhan, China

**Keywords:** Cell division, Cell signalling

## Abstract

The immune system is finely tuned to fight against infections, eradicate neoplasms, and prevent autoimmunity. Protein posttranslational modification (PTM) constitutes a molecular layer of regulation to guarantee the proper intensity of immune response. Herein, we report that UBC9-mediated protein SUMOylation plays an essential role in peripheral CD4 T-cell proliferation, but without a perceptible impact on T-cell polarization. Both conventional T-cell (Tcon) and regulatory T-cell (Treg) maintenance are differentially affected, which was likely caused by a shared deficit in cell glycolytic metabolism. Mechanistically, PDPK1 (3-phosphoinositide-dependent protein-kinase 1) was identified as a novel SUMOylation substrate, which occurred predominantly at lysine 299 (K299) located within the protein-kinase domain. Loss of PDPK1 SUMOylation impeded its autophosphorylation at serine 241 (S241), thereby leading to hypoactivation of downstream mTORC1 signaling coupled with incompetence of cell proliferation. Altogether, our results revealed a novel regulatory mechanism in peripheral CD4 T-cell homeostatic proliferation, which involves SUMOylation regulation of PDPK1–mTORC1 signaling-mediated glycolytic process.

## Introduction

CD4 T cells are fundamental for the initiation of proper immune response and the maintenance of tissue homeostasis [1]. In the thymic compartment, bone marrow-derived common lymphoid progenitor cells (CLP) undergo multi-step developmental processes, including DN1–DN4 (double-negative) transition, DP (double-positive) cell formation after positive selection, and the final CD4 SP (single-positive) stage following negative selection. During the negative-selection processes, some matured cells are committed to regulatory T (Treg) cells, while the others make up the conventional T- (Tcon) cell population [2]. However, homeostatic proliferation is critical to maintain the functional and diverse pool of thymus-emigrant lymphocytes in the periphery, which is finely coordinated to ensure immune defense and self-tolerance [3, 4].

Glucose metabolism and glycolysis have long been recognized to engage in both CD4 T-cell development and the homeostatic proliferation of matured cells [5]. External stimuli activate PI3K/AKT signaling and promote the expression of glycolytic genes such as Eno1, PGK1, and glucose transporter Glut1, thereby bolstering the energy-consuming metabolic process [6]. The mammalian target of rapamycin (mTOR) is an important intermediary signaling molecule manifesting at least two complexes, mTORC1 and mTORC2 [7]. mTORC2, featured by the core components Rictor and Sin1, is implied in the early DN stage of T-cell development, while the DP and CD4 SP cells are unperturbed by mTORC2 ablation [8]. The promigratory stimuli activate the PI3K–mTORC2 pathway via CD28 and LFA-1 to regulate cytoskeletal rearrangements by associating actin in nonproliferating Treg cells [9]. In contrast, mTORC1, consisting of the signature constituent Raptor, plays a pivotal role in CD4 T-cell differentiation, activation, and proliferation after its maturation [10, 11]. Particularly, coordinated mTORC1 signaling is essential to maintain Treg stability and its suppressive function [12–17].

The 3-phosphoinositide-dependent kinase 1 (PDPK1), a pleckstrin domain containing protein kinase, is a master regulator of AGC Ser/Thr kinases, including PKB/AKT, PKC, S6K, and SGK [18, 19], which are tightly associated with glycolytic metabolism. Therefore, loss of PDPK1 blocks the developing thymocytes at the DN4 stage. Intriguingly, PDPK1 deficiency in DP thymocyte does not affect CD4 SP, CD8 SP, and Treg generation in the thymus, nor the lineage commitment of the matured cells, but is coupled with a substantially reduced CD4 T-cell number in the periphery [20–22]. However, mice with CD4 T-cell-specific PDPK1 deficiency exhibit unexpected autoimmune response associated with Treg impairment over minor Tcon defect [23, 24]. In general, PDPK1 activation involves its binding to PIP3, a product of PI3K-catalyzed conversion of PIP2, by which it translocates to the membrane [25]. Nevertheless, there is also feasible evidence that PDPK1 can exert its function in a PI3K-independent manner [26]. Therefore, the mechanisms underlying PDPK1 activation, particularly its posttranslational modification (PTM) regulatory machinery, are yet to be fully addressed.

UBC9-mediated SUMOylation has been reported to be dynamically involved in T-cell biology, which involves substrates related to either downstream of TCR signaling (e.g., PKC-θ, IκB, NFAT1, and JunB) or T-cell differentiation (e.g., c-MAF, RORγ-T) [27, 28]. Particularly, SUMOylation of IRF4 plays a critical role in Treg specialization and effector activity [29], while UBC9 deficiency impairs normal thymus developmental process [30]. However, the impact of SUMOylation on the homeostasis of peripheral Tcon and Treg cells remains elusive. Herein, we report that deficiency of PDPK1 SUMOylation abrogates its kinase activity and leads to impaired glycolytic pathway. As a result, the proliferation of peripheral CD4 T cells, and Treg cells in particular, is significantly abrogated. Our work identifies that SUMOylation of PDPK1 acts as an essential regulatory mechanism to maintain the homeostasis in peripheral CD4 T cell.

## Results

### Loss of Ubc9 impairs CD4 T-cell proliferation along with lymphoid-organ atrophy

**Fig. 1:**
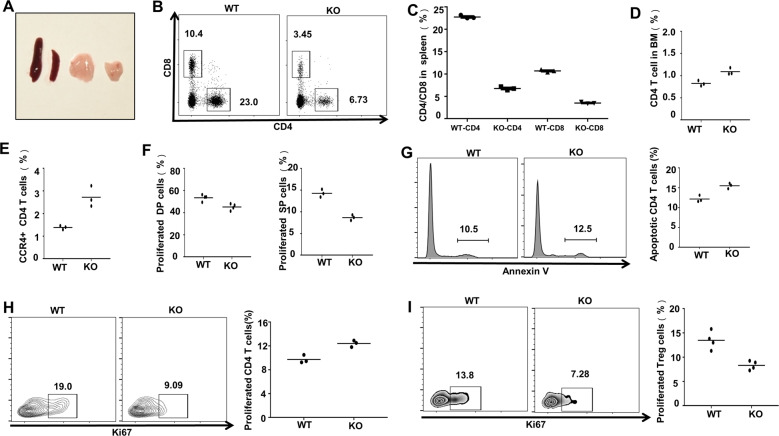
Loss of Ubc9 impairs CD4 T cell proliferation along with lymphoid organ atrophy. **A** Representative photograph of thymus and spleen from WT (left) and KO (right) 5-week-old mice. **B**, **C** Spleens were surgically removed to determine the peripheral CD4 and CD8 T cells. Proportion of splenic CD4 T cells: (WT: 22.77 ± 0.22% vs. KO: 6.76 ± 0.23%, *p* < 0.001); CD8 T cells: (WT: 10.70 ± 0.17% vs. KO: 3.52 ± 0.11%, p < 0.001). Count of splenic CD4 T cells: (WT: 9.78 ± 0.36 × 10^6^ vs. KO: 1.48 ± 0.20 × 10^6^, *p* < 0.001). **D** Bone marrow (BM) cells were flushed to check for CD4 T-cell percentage within the BM compartment, and (**E**) the expression of CCR4 was examined on peripheral CD4 T cells. **F** Thymuses were taken to probe the Ki67^+^ proliferative cells in either DP (WT: 53.53 ± 1.46% vs. KO: 45.18 ± 1.47%, *p* < 0.01) or CD4 SP (WT: 14.27 ± 0.52% vs. KO: 8.66 ± 0.36%, *p* < 0.001) thymocytes. **G** Annexin-V staining was applied to detect the apoptosis level of peripheral CD4 T cells (WT: 9.73 ± 0.39% vs. KO: 45.18 ± 1.47%, *p* < 0.01). **H**, **I** CD4 T cell (WT: 18.17 ± 0.78% vs. KO: 10.30 ± 1.25%, *p* < 0.01) and Treg proliferation (WT: 13.48 ± 0.93% vs. KO: 8.31 ± 0.46%, *p* < 0.01) were indicated by Ki67 positively stained cells. For (**B**–**H**), the mean ± SD is shown from *n* = 3 mice. For (I), the mean ± SD is shown from *n* = 5 mice. The *p*-value was determined by Student’s unpaired *t*-test.

We first generated CD4 T-cell-specific Ubc9-knockout mouse model by crossing the Ubc9^fl/fl^ mice with Cd4^Cre^ mice, and the resulting Cd4^Cre^–Ubc9^fl/fl^ mice were denoted as KO thereafter. It was interestingly noted that the KO mice manifested smaller size for both spleen and thymus (Fig. 1A). The proportion of peripheral CD4 T cells declined by 2- to 3-fold (Fig. 1B, C), and the cell number declined by 6- to 7-fold (Fig. S2A), but the KO mice were absent of dry-eye and scurfy-skin symptoms. An increased retention of CD4 T cells in the bone marrow (Fig. 1D, Fig. S2B) along with enhanced surface expression of periphery-homing receptor CCR4 (Fig. 1E) was also noted, which may contribute marginally to the diminished CD4 T cells in the lymphoid organ.

Since a 2-fold reduction of CD8 T cells was also observed (Fig. 1C), we then checked apoptosis and proliferation of thymocytes in the thymic compartment, which may contribute to the observed phenotype. No perceptible difference regarding T-cell developmental stages (Fig. S1A, B) was detected, and the apoptotic thymocyte ratio was also comparable between DP cells, despite that the KO SP cells showed around 1-fold decrease of apoptosis (Fig. S1C). In contrast, the Ki67^+^ proliferated thymocytes were significantly reduced at both DP and SP stage, which explains the concurrent reduction of CD8 T cells (Fig. 1F). Although the mature KO CD4 T cells exhibited slightly higher apoptosis rate (Fig. 1G), they displayed significantly impaired proliferation capability (Fig. 1H). The proportion of proliferated Treg cells also declined by around 1-fold (Fig. 1I), and notably, the naturally occurring Nrp1^+^ subset (nTreg) rather than peripherally induced Nrp1^−^ counterpart (pTreg) was mostly affected as viewed by the decrease of nTreg/pTreg ratio (Fig. S1D). Collectively, those data suggest that loss of SUMOylation function abrogates the proliferative capacity of CD4 T cells, which likely contributes to lymphoid-organ atrophy.

### Ubc9 deficiency manifests distinctive effect on peripheral T-cell subsets

**Fig. 2:**
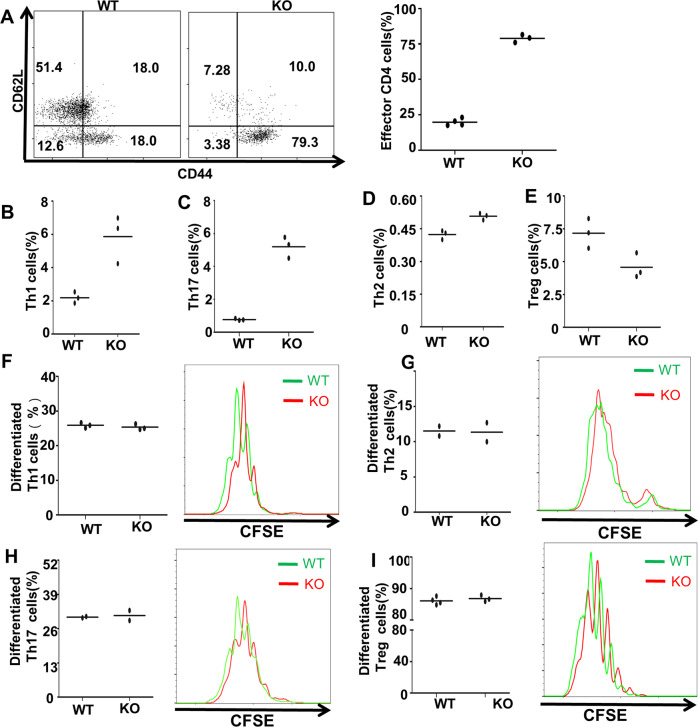
Ubc9 deficiency manifests distinctive effect on peripheral T-cell subsets. **A** Shown is the CD44^+^ CD62L^lo^ activated effector T cells (*n* = 3 mice per group) in peripheral CD4 T cells (WT: 19.80 ± 1.27% vs. KO: 78.93 ± 1.63%, *p* < 0.001). **B**, **C** Effector T-cell subsets, including Th1 (WT: 2.19 ± 0.20% vs. KO: 5.86 ± 0.83%, *p* < 0.05), Th17 (WT: 0.77 ± 0.04% vs. KO: 5.20 ± 0.37%, *p* < 0.001), and Th2 (WT: 0.42 ± 0.01% vs. KO: 0.51 ± 0.01%, *p* < 0.01) were determined by flow cytometry (*n* = 3). **D** Treg cell percentage (WT: 7.17 ± 0.65% vs. KO: 4.57 ± 0.56%, *p* < 0.05) was examined in peripheral CD4 T cells (*n* = 4). **E**–**H** CD4 naive T cells were labeled with CFSE, and then differentiated under Th1, Th2, Th17, and Treg conditions for 3–5 days. Th1: WT: 25.75 ± 0.25% vs. KO: 25.65 ± 0.65%, *p* = 0.90; Th2: WT: 11.50 ± 0.70% vs. KO: 11.35 ± 1.35%, *p* = 0.93; Th17: WT: 30.35 ± 0.25% vs. KO: 31.00 ± 1.90%, *p* = 0.77; and Treg: WT: 86.55 ± 1.05% vs. KO: 86.90 ± 1.00%, *p* = 0.83. Differentiation efficiency and proliferation were shown in the bar graph and flow histograms (2-times biological replication for Th2 and Th17; 3-times biological replication for Th1 and Treg). The *p*-value was determined by Student’s unpaired *t-*test.

To confirm that loss of SUMOylation function impacts the proliferative capacity of peripheral CD4 T-cells, we first checked the effector T cell subsets in the KO mice. It was found that CD4^+^ CD44^+^ CD62L^lo^ effector-memory T cells (T_EM_) increased dramatically in the KO CD4 T cells (Fig. 2A), while the KO mice exhibited a significant increase for the IFN-γ^+^ Th1 cells (Fig. 2B) and IL-17A^+^ Th17 cells (Fig. 2C), along with a slight increase of IL-4^+^ Th2 cells (Fig. 2D). In sharp contrast, the proportion of Treg subset decreased by almost 50% (Fig. 2E), suggesting that Ubc9 deficiency probably induces higher severity of impairment on Treg cells as compared with Tcon cells. However, in terms of the absolute number, all Th subsets exhibited a decrease of cell counts (Fig. S2C, D, F), except for Th17 (Fig. S2E), which further implied the deficit of CD4 T-cell homeostasis.

Naive CD4 T cells were isolated and then labeled with cell-trace dye CFSE before stimulation under Th1, Th2, Th17, and Treg conditions as described. Surprisingly, we failed to detect a discernable difference in terms of T-cell polarization between the WT and KO naive CD4 T cells, although the proliferation of helper T-cell subsets was impaired in KO cells (Fig. 2F–I). Taken together, our data support that loss of SUMOylation function selectively impairs the proliferative capacity of CD4 T cells rather than disturbing their polarization program.

### Ubc9 deficiency intrinsically affects peripheral T-cell development

**Fig. 3:**
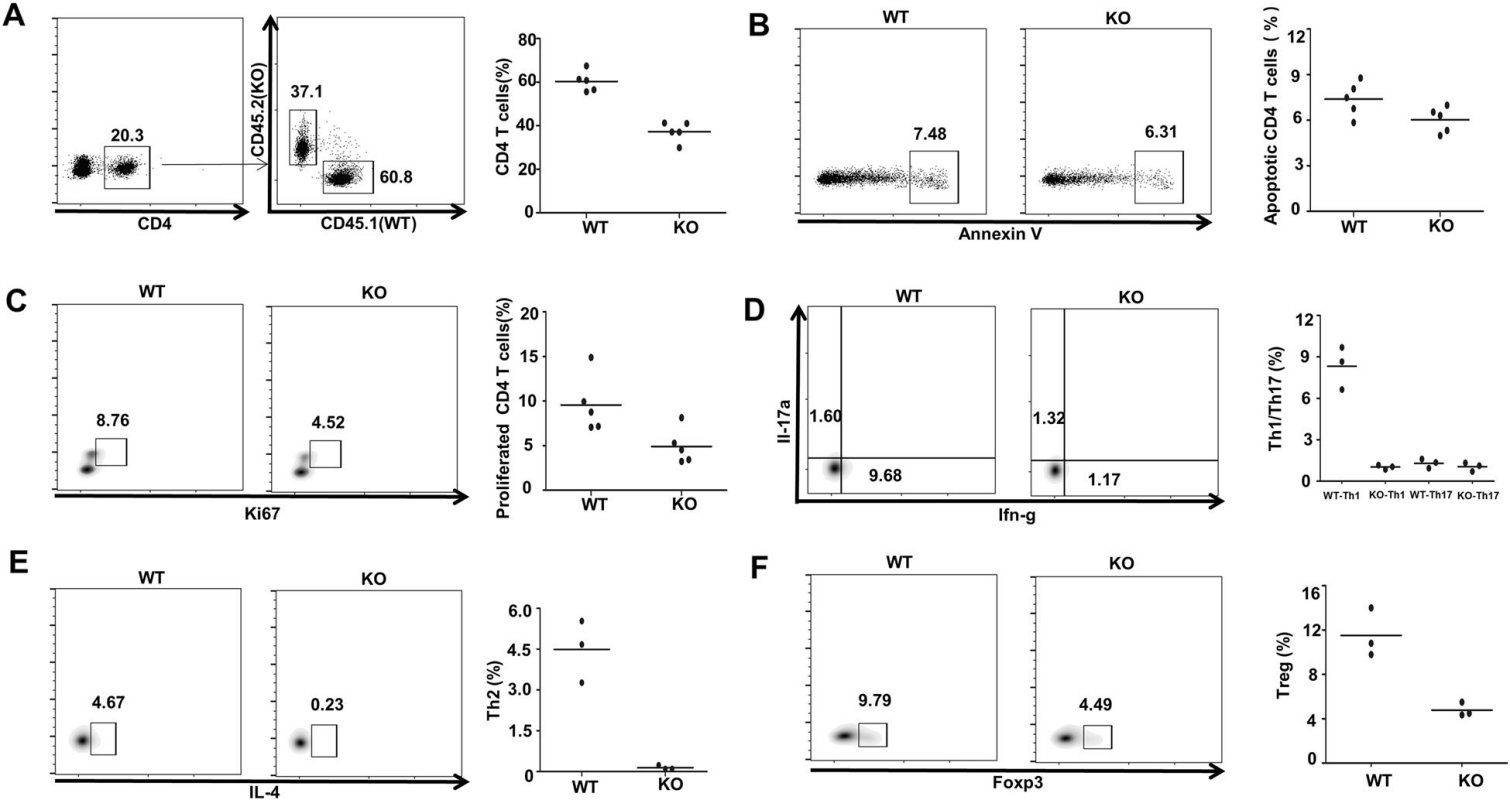
Ubc9 deficiency intrinsically affects peripheral T-cell development. Recipient CD45.1 (WT) mice (*n* = 5) were lethally irradiated (1000–1100 cGy, two split doses, 4 h apart) and each mouse was intravenously injected 1 × 10^7^ total mixed BM cells from CD45.1 (WT) and CD45.2 (KO) mice at the ratio of 1:1. The mice were sacrificed for further analysis eight weeks after the transplantation. **A** Occupation of WT and KO cells was determined in CD4 gate. **B**, **C** Apoptosis (WT: 7.39 ± 0.51% vs. KO: 6.03 ± 0.38%, *p* = 0.06) and proliferation (WT: 9.56 ± 1.44% vs. KO: 4.91 ± 0.89%, *p* < 0.05) of CD4 T cells from different sources were examined. **D**–**F** Detection of various T-cell subsets was conducted by flow-cytometry analysis. Th1: WT: 8.33 ± 0.89% vs. KO: 1.02 ± 0.09%, *p* < 0.01; Th2: WT: 4.49 ± 0.66% vs. KO: 0.14 ± 0.04%, *p* < 0.01; Th17: WT: 1.30 ± 0.19% vs. KO: 1.05 ± 0.18%, *p* = 0.39; Treg: WT: 11.53 ± 1.27% vs. KO: 4.78 ± 0.36%, *p* < 0.01. The *p*-value was determined by Student’s unpaired *t*-test.

The chimeric bone marrow adoptive-transfer (BMT) model was next employed to further address the impact of Ubc9 deficiency on peripheral CD4 T cells. Bone marrow from CD45.2^+^ KO mice and CD45.1^+^ WT mice was mixed at a 1:1 ratio, and then adoptively transferred into sublethal dose of X-ray- (1100 cGy) irradiated recipient mice. The mice were sacrificed for analysis of key immune signatures eight weeks later. As expected, relatively low enrichment ratio of KO CD4 T cells (~40%) was observed in the peripheral immune compartment, indicating that WT cells outcompetesKO ones within the same host (Fig. 3A). We then checked apoptosis and proliferation. Similarly, we failed to detect an obvious difference in terms of apoptosis between groups (Fig. 3B). However, Ki67 staining revealed a defect in KO CD4 T-cell proliferation (Fig. 3C), which likely accounts for the relative low abundance of KO CD4 T-cell occupation.

Interestingly, a distinct profiling from different T-cell sources was noted. Specifically, Th17 cells did not show much difference (Fig. 3D). WT-derived CD4 T cells retained normal Th1 and Th2 frequency, but KO-derived Th1 (Fig. 3D) and Th2 (Fig. 3E) lineages showed a significant reduction, and Th2 cells in particular, were almost undetectable in mice transferred with KO bone marrow (Fig. 3E). Unlike the incongruity of Tcon subsets observed in KO mice (Fig. 2B, C), the proportion of Treg cells displayed a consistent decrease (Fig. 3F). These results suggest that the deficit of peripheral effector T cells could be also a consequent event resulted from Treg reduction in the KO mice.

### Ubc9 deficiency impairs the homeostatic maintenance of Treg cells

**Fig. 4:**
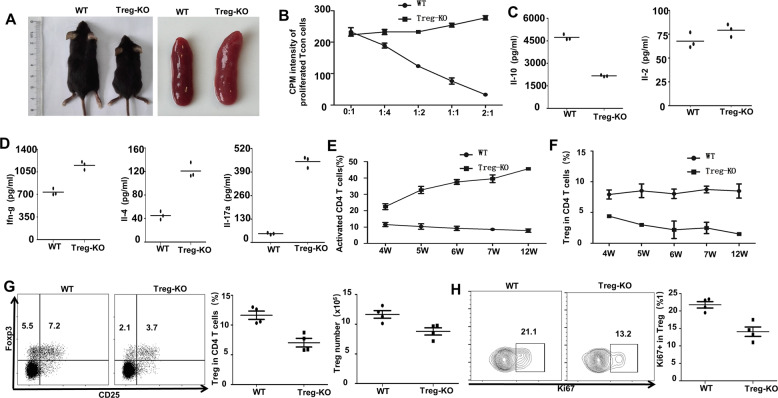
Ubc9 deficiency impairs the homeostatic maintenance of Treg cells. **A** Representative photograph of mouse size and spleen from WT (left) and Treg-KO (right) 5-week-old mice. **B** Treg cells were isolated and cocultured with Tcon cells at different ratio (Treg/Tcon = 0:1, 1:4, 1:2, 1:1, and 2:1). Proliferation of Tcon cells was indicated by the intensity of [3][H] incorporation, which was negatively correlated with Treg suppressive function (*n* = 3, representative of two experiments). **C**, **D** CD4^+^CD25^+^ or CD4^+^CD25^−^ T cells were isolated and stimulated by anti-CD3 (10ug/ml) for 3 days, supernatants were collected for the detection of cytokine concentration of IL-10 (WT: 4727 ± 108.7 pg/ml vs. Treg-KO: 2167 ± 31.51 pg/ml, *p* < 0.001), IL-2 (WT: 67.80 ± 4.73 pg/ml vs. Treg-KO: 79.32 ± 3.84 pg/ml, *p* = 0.13), IFN-γ (WT: 735.7 ± 29.36 pg/ml vs. Treg-KO: 1,152 ± 36.57 pg/ml, *p* < 0.001), IL-4 (WT: 45.28 ± 4.04 pg/ml vs. Treg-KO: 121.0 ± 7.35 pg/ml, *p* < 0.001), and IL-17A (WT: 48.71 ± 3.06 pg/ml vs. Treg-KO: 447.5 ± 17.76 pg/ml, *p* < 0.001) (*n* = 3). **E**, **F** Time-dependent increase of activated T cells and decrease of Treg cells are shown. **G** Proportion (WT: 11.64 ± 0.69% vs. Treg-KO: 7.03 ± 0.72%, *p* < 0.01) and absolute number (WT: 11.67 ± 0.63 × 10^5^ vs. Treg-KO: 8.78 ± 0.61 × 10^5^, *p* < 0.05) of Treg cells in 4–5-week-old WT and Treg-KO mice (*n* = 4). **H** Percentage of Ki67^+^ proliferative Treg cells (WT: 21.78 ± 0.91% vs. Treg-KO: 14.08 ± 1.36%, *p* < 0.01) in 4–5-week-old WT and Treg-KO mice (*n* = 4). The *p*-value was determined by Student’s unpaired *t*-test.

To confirm the above assumption, we generated Treg-specific Ubc9 KO mice (defined as Treg-KO mice thereafter) by crossing the Foxp3^cre^ mice with the Ubc9^fl/fl^ mice as above. Unsurprisingly, knockout mice demonstrated scurfy-like manifestations and splenomegaly (Fig. 4A). In vitro-suppressive assay showed abrogated Treg suppressive function (Fig. 4B), and the content of IL-10 decreased by more than 1-fold in the CD4^+^ CD25^+^ Treg-KO cells (Fig. 4C). Unlike the results observed in the bone marrow chimeric mice (Fig. 3D, E), the effector cytokines such as IFN-γ, IL-4, and IL-17A were significantly increased in CD4^+^ CD25^−^ cells (Fig. 4D). Indeed, flow cytometry analysis revealed elevated activated T cells (Fig. 4E) along with decreased Treg cells (Fig. 4F) in peripheral blood in an age-dependent manner, which corroborated the impaired proliferation of Treg cells. We then checked splenic Treg cells in 4- to 5-week-old mice. Consistently, compared with WT mice, Treg-KO mice harbored reduced Treg proportion and cell number (Fig. 4G). Meanwhile, the Ki67^+^ proliferative Treg cells in Treg-KO mice also decreased at this early age (Fig. 4H). Together, those data support that loss of SUMOylation function impairs the homeostatic maintenance of Treg cells, thereby leading to the activation of autoreactive immune cells.

### Ubc9 deficiency impairs PDPK1 signaling coupled with altered glycolysis

**Fig. 5:**
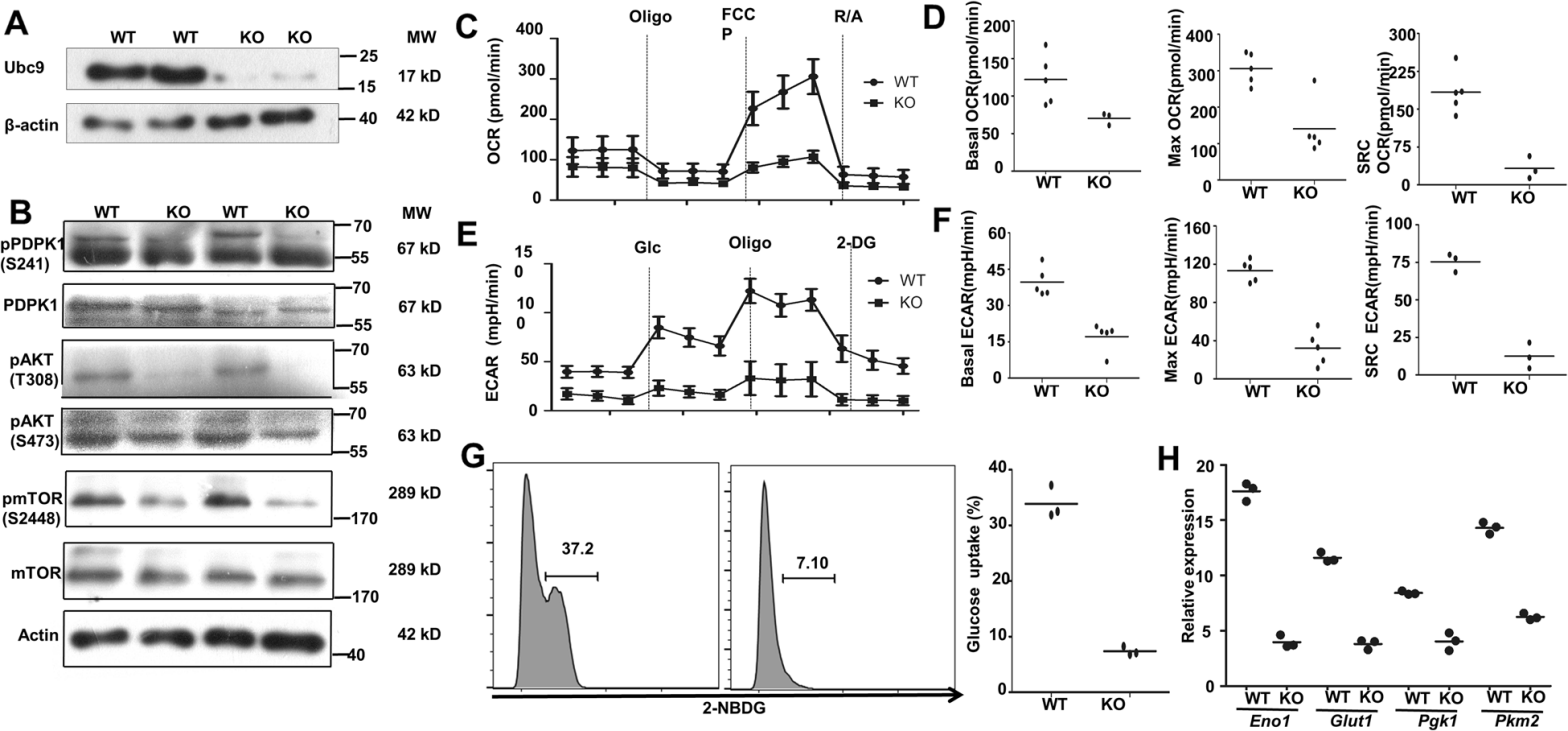
Ubc9 deficiency impairs PDPK1 signaling coupled with altered glycolysis. **A**, **B** Expression levels of UBC9, (p-)PDPK1, (p-)AKT, and (p-)mTOR as detected by Western blot. **C** OCR of control and KO CD4 T cells at basal levels, followed by sequential treatment (dashed lines) of oligomycin (Oligo), FCCP, and rotenone plus antimycin (R/A) (representative of two experiments). **D** Accordingly, baseline OCR (WT: 122.0 ± 14.87 pmol/min vs. KO: 70.34 ± 4.69 pmol/min, *p* < 0.05) (WT: *n* = 5, KO: *n* = 3), maximal respiration OCR (WT: 305.7 ± 19.29pmol/min vs. KO: 140.3 ± 33.70 pmol/min, *p* < 0.01) (WT: *n* = 5, KO: *n* = 5), and the reserved OCR capacity (WT: 183.7 ± 19.05 pmol/min vs. KO: 32.60 ± 12.82 pmol/min, *p* < 0.01) (WT: *n* = 5, KO: *n* = 3) were shown. **E** ECAR of control and KO CD4 T cells at basal levels, followed by sequential treatment (dashed lines) of glucose (Glc), Oligo and 2-DG (representative of two experiments). **F** Accordingly, baseline glycolysis (WT: 39.65 ± 2.68 mpH/min vs. KO: 17.13 ± 2.61 mpH/min, *p* < 0.001) (*n* = 5), maximal glycolytic capacity (WT: 113.2 ± 4.99 mpH/min vs. KO: 32.15 ± 7.89 mpH/min, *p* < 0.001) (*n* = 5), and glycolytic reserve (WT: 75.42 ± 3.56 mpH/min vs. KO: 12.54 ± 4.94 mpH/min, *p* < 0.001) were shown (*n* = 3). **G** Glucose-uptake assay was performed to measure the ability of glucose usage by WT and KO CD4 T cells (WT: 33.87 ± 1.68% vs. KO: 7.39 ± 0.44%, *p* < 0.001) (*n* = 3). **H** Quantification of the relative mRNA abundance of key glycolytic genes (Eno1, Glut1, Pgk1, and Pkm2) was determined by RT-PCR (*n* = 3, representative of two experiments). The *p*-value was determined by Student’s unpaired *t*-test.

Since CD4 T-cell-specific PDPK1 deletion induces lymphoid atrophy, and PDPK1 deficiency in Treg cells generates severe spontaneous autoimmune diseases [31], which resemble the phenotypes observed in our KO and Treg-KO mice, we then examined PDPK1 and its downstream signaling molecules. We first confirmed that Ubc9 was depleted in CD4 T cells isolated from the KO mice (Fig. 5A). Remarkably, a significantly decreased PDPK1 phosphorylation was noted in peripheral Ubc9 deficient CD4 T cells (Fig. 5B), which prompted us to check its downstream AGK kinases, AKT and mTORC1. Consistently, markedly reduced levels for phosphorylated AKT (pAKT) at T308 and p-mTORC1 at S2448 were detected in Ubc9-deficient CD4 T cells, and in contrast, the phosphorylation of S473 in AKT, which is relevant to mTORC2 signaling, did not show a perceptible difference (Fig. 5B). Furthermore, decreased phosphorylation of S6K was detected in Ubc9 deficient CD4 T cells and Treg cells (Fig. S3A, C), and the downregulated PDPK1 phosphorylation was also confirmed in Treg-KO cells (Fig. S3B).

As aforementioned, PDPK1 plays a critical role in glycolytic process [26] to provide building blocks for cell division and the subsequent generation of daughter cells. We, thus next, conducted metabolic assays in Ubc9-deficient CD4 T cells. The oxygen-consumption rate (OCR) was measured to determine the mitochondrial respiration (Fig. 5C). The KO cells exhibited lower basal mitochondrial OCR, lower maximal respiration capability along with decreased spared respiratory capacity (SRC) (Fig. 5D). Importantly, severely attenuated glycolytic process evidenced by the reduction of extracellular acidification rate (ECAR) was characterized (Fig. 5E). Specifically, the basal glycolytic rate was only 40% of its WT control coupled with a marked impairment of the maximal glycolysis capacity following oligomycin induced mitochondria inhibition and SRC (Fig. 5F). Overall, compared with mitochondrial respiration, the glycolytic process was predominantly affected, even under the basal condition. Glucose-uptake assays using the fluorescence labeled 2-NBDGT were then employed to confirm these findings. Indeed, the KO CD4 T cells exhibited reduced glucose-uptake activity (Fig. 5G) along with reduced expression of pivotal glycolytic genes, including Eno1, Glut1, PGK1, and PKM2 (5H). Taken these data together, our results support that Ubc9 deficiency impairs PDPK1 signaling, which disrupts glycolytic process to attenuate CD4 T-cell proliferation.

### UBC9 modulates PDPK1 activity by mediating its SUMOylation

**Fig. 6:**
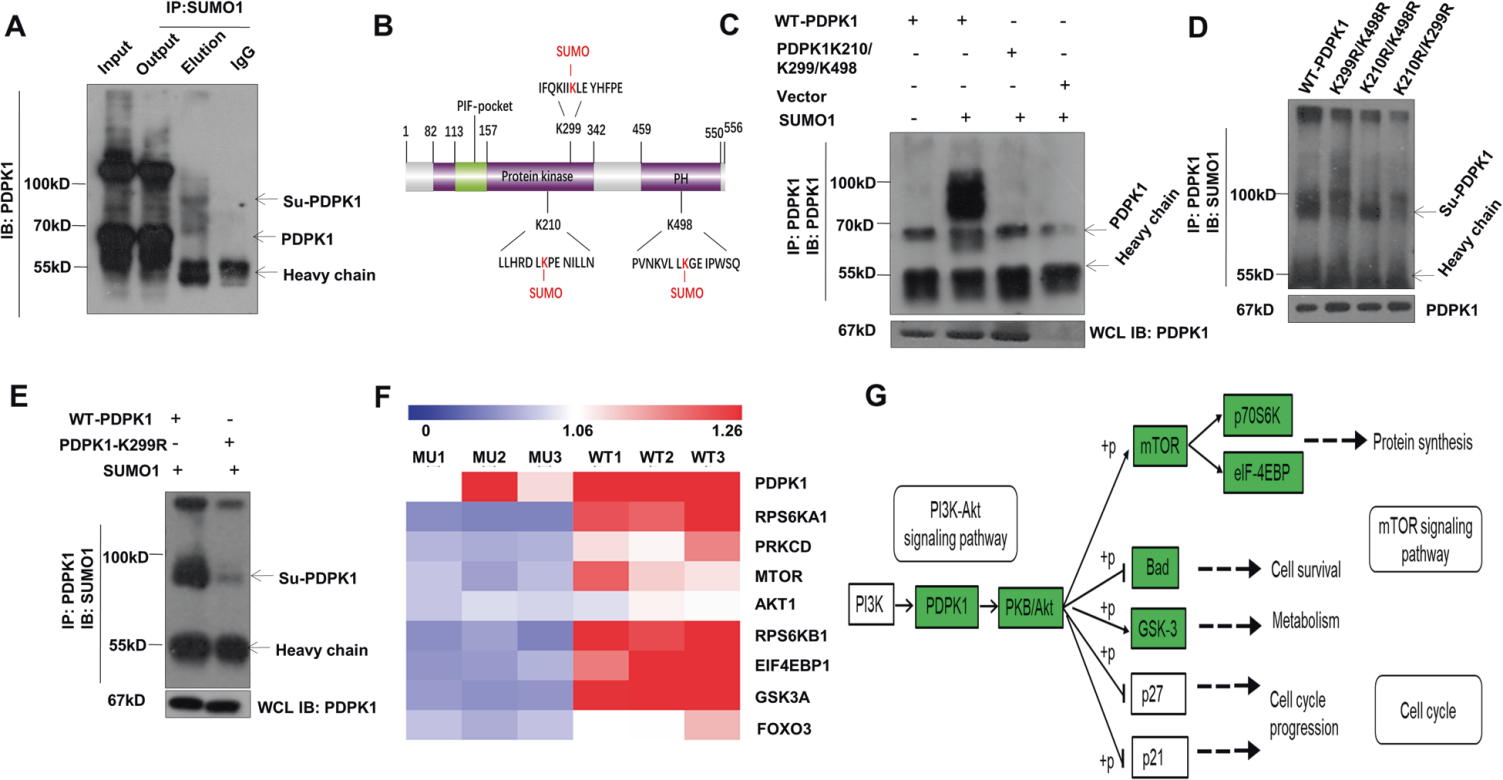
UBC9 modulates PDPK1 activity by mediating its SUMOylation. **A** CD4 T cells were immune-precipitated by SUMO1 and probed with PDPK1. Band shift of PDPK1 SUMOylation (Su-PDPK1, top arrow) was observed below 100KD (representative of 5 times result). **B** Schematic view of SUMOylation sites within protein-kinase domain and PH domain. **C** Triple PDPK1 mutants (K210/K299/K498) confirmed the SUMOylation sites (representative of 2-times result). **D**, **E** Series of double mutants (K210/K299, K210/K498, and K299/K498) and K299 single mutant identified K299 as the major SUMOylation site (representative of 3-times result). **F**, **G** Phospho-proteomic study identified Mu/WT downregulated phosphoproteins that are downstream of PDPK1 and related to mTORC1 signaling, as indicated by Heatmap and KEGG pathway enrichment analysis.

To dissect the mechanisms by which UBC9 modulates PDPK1 activity, we first examined PDPK1 SUMOylation. CD4 T cells were subjected to co-immunoprecipitation using polyclonal SUMO1 antibody and probed by PDPK1. Clearly, a PDPK1 reactive band with higher molecular weight was detected in the elution fraction (<100KD, >70 Kd) (Fig. 6A). Bioinformatic prediction narrowed the candidate sites to K210 and K299 located within the protein-kinase (PK) domain, and K498 within the pleckstrin-homology (PH) domain (Fig. 6B). Multiple-site mutagenesis was then conducted to generate triple-mutated plasmid, in which the lysine residues (K) were mutated to arginine (R). The plasmid was next cotransfected with SUMO1 into HEK293T cells. Consistently, the SUMOylated band could be only detected in wild-type plasmid-transfected cells (Fig. 6C). To discriminate the major site(s) from minor site(s), we then constructed a combination of double-mutated plasmids (K299R/K498R, K210R/K498R, and K210R/K299R). It was noted that K299 is likely the predominant SUMOylation target, while K210 and K498 have a marginal contribution (Fig. 6D). Transfection of K299R alone further confirmed the conclusion, as evidenced by the detection of a very faint band once K210 and K498 were preserved (Fig. 6E). To get rid of potential SUMOylation activity, hereafter, triple-mutation construct was applied for the following experiments.

Since K210 and K299 are located within the PK domain (Fig. 6B), we then checked SUMOylation on PDPK1 kinase activity by phosphoproteomic analysis. HEK293T cells were transfected with an equal amount of WT or MU plasmid, then lysed for mass spectrometry analysis. The level and modification sites of differentially phosphorylated proteins (MU/WT) were listed in Supplementary Table 1. Gene-ontology analysis unveiled the critical involvement of proteins related to metabolic process and the catalytic activity (Fig. S2A, B). In addition, motif analysis showed that upregulated phosphorylation mainly occurs on nucleotide binding domain and downregulated phosphorylation occurs on PH-domain-like and pleckstrin-homology domain (Fig. S2C, D). Strikingly, the phosphorylation of PDPK1 and its downstream molecules relevant to glycolysis, such as RPS6K, mTOR, and 4EBP1, were downregulated in the MU plasmid-transfected cells (Fig. 6F). Moreover, KEGG pathway-enrichment analysis revealed that these key downregulated proteins were situated in the mTORC1 signaling pathway (Fig. 6G), which further confirmed our hypothesis.

### SUMOylation of PDPK1 is essential for CD4 T cell proliferation

**Fig. 7:**
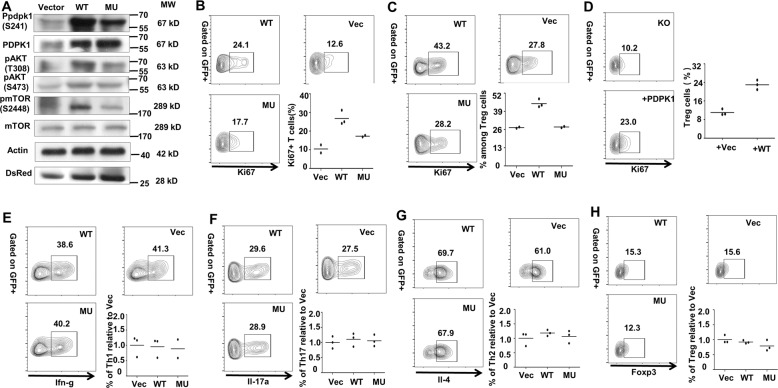
SUMOylation of PDPK1 is essential for CD4 + T-cell proliferation. **A** Transfection of WT and MU PDPK1 confirmed the phospho-proteomic result, as indicated by the expression of (p-) PDPK1 (Ser241), (p-) AKT (Thr308), (p-) AKT (Ser403), and (p-) mTOR (Ser2448) (3 times replication). **B** Naive T cells were transduced with WT (*n* = 3) and MU PDPK1 (*n* = 2) virus. Shown is the Ki67^+^ proliferative cells within each group (WT: 26.77 ± 2.23% vs. MU: 17.30 ± 0.40%, *p* < 0.05). **C** Treg cells were transduced with WT and MU PDPK1 virus. Shown is the Ki67^+^ proliferative cells within each group (WT: 44.53 ± 1.71% vs. MU: 27.75 ± 0.45%, *p* < 0.01) (*n* = 3). **D** Ubc9-deficient Treg cells were transduced with either vector or WT PDPK1 virus. Shown is the Ki67^+^ proliferative cells within each group (*n* = 3). **E**–**H** Naive T cells were transduced with WT and MU PDPK1 virus, and differentiated into distinct subsets. For MU Th1, (*n* = 2); for rest of the conditions, (*n* = 3). The *p*-value was determined by Student’s unpaired *t*-test.

Next, we conducted experiments to confirm the above proteomic results. Indeed, the phosphorylation of PDPK1 (S241), AKT (T308), and mTORC1 (S2448) was decreased in cells transfected with mutant PDPK1, while AKT (S473) related to mTORC2 signaling did not show much difference (Fig. 7A). ECAR analysis by Seahorse revealed an ablated glycolytic metabolism in MU PDPK1-transfected cells (Fig. S5A). Specifically, the basal glycolytic rate in MU PDPK1 group was around 60% of the WT PDPK1 control coupled with a marked impairment of the maximal glycolysis capacity and SRC (Fig. S5B–D). We then sought to address the direct impact of PDPK1 SUMOylation on CD4 T cell proliferation. Naive CD4 T-cells were transduced with lentiviral particles containing vector (Vec), wild type (WT), or mutant (MU) PDPK1 as described, and then subjected to analysis of Ki67^+^ proliferative cells. In line with our expectation, the proportion of Ki67^+^ cells in the MU group was much less than the WT controls, and cells in the Vec group were featured by the lowest ratio (10.47 ± 2.14%) (Fig. 7B). Similarly, Treg cell-transduced WT viruses displayed a 50% higher proliferative ratio as compared with cells in the MU group (Fig. 7C). Since much higher severity of proliferative deficit was observed in Treg cells, we employed Treg cells for the rescue assays. Indeed, reintroduction of PDPK1 restored the proliferation capacity in Ubc9 KO Treg cells (Fig. 7D).

Finally, we intended to demonstrate that loss of PDPK1 SUMOylation does not affect CD4 T-cell differentiation (T-cell polarization program), which was not caused by the global ablation of SUMOylation function. For this purpose, naive CD4 T cells were transduced with indicated PDPK1 lentiviruses and then cultured them under polarizing conditions as described, respectively. As expected, deficiency of PDPK1 SUMOylation did not affect Th1 (Fig. 7E), Th17 (Fig. 7F), and Th2 (Fig. 7G) polarization, and similar results were noted in Treg polarization as well (7H). Overall, these results support that SUMOylation of PDPK1 does not affect CD4 T-cell polarization program, but is essential to their proliferation.

## Discussion

**Fig. 8:**
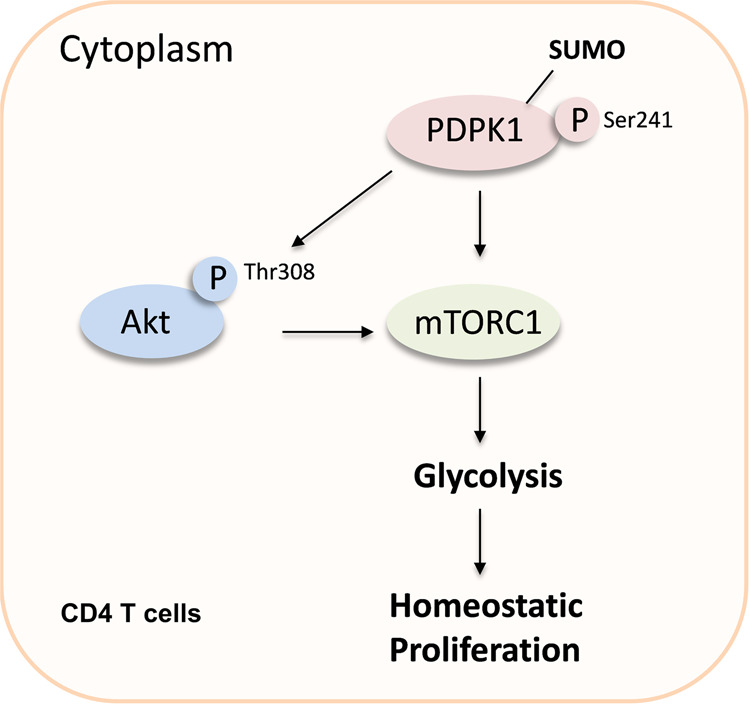
Graphical illustration. SUMOylation of PDPK1 regulates its kinase activity and downstream signaling, thereby modulating glycolysis-dependent CD4 T-cell homeostatic proliferation.

Previously, our group demonstrated the pivotal role of UBC9 in the regulation of pancreatic beta-cell viability and functionality, macrophage polarization, and common lymphoid-progenitor cell (CLP) expansion [32–34]. In this study, we provided convincing evidence indicating a role of UBC9-mediated SUMOylation in the maintenance of peripheral CD4 T-cell homeostasis, in which SUMOylation of PDPK1 regulates its kinase activity and downstream signaling, thereby modulating glucose glycolysis essential for CD4 T-cell proliferation (Fig. 8).

Mice with Ubc9 deficiency in CD4 T cells manifested significantly reduced CD4 T cells in the periphery while exhibited more activated phenotype. However, scurfy-like autoimmune symptoms were absent, but the mice displayed signs of lymphoid atrophy. Although the KO mice displayed growth retardation during their early age (4–5 weeks), but they could slowly catch up with their littermates in terms of body size at around 12 weeks of age. Deficit of proliferation other than apoptosis is thought mostly contributing to the decline of peripheral CD4 T cells, which occurs both in Treg and Tcon cells under physiological condition. Intriguingly, we detected increased Th1, Th2, and Th17 ratio among peripheral CD4 T cells, suggesting an imbalance of Treg/Tcon even though both of which declined in absolute cell count. Upon activation, CD4 T cells upregulate the expression of various chemokine receptors that facilitate their egress from circulation and retention in the bone marrow (e.g., CXCR4) and peripheral tissues (e.g., CCR4) [35]. Indeed, we found an increased presence of CD4 T cells in the bone marrow and elevated expression of CCR4 in the peripheral CD4 T cells. Nonetheless, comparing to the dramatic reduction of cell proliferation, the contribution of cell migration may be very limited.

Treg and Tcon are two counteracting and complementary cell subsets. Overactive Tcon would secrete inflammatory cytokines and dampen the stability and suppressive capability of Treg cells. On the other hand, Treg functional adaptation and homeostatic proliferation rely on IL-2 secreted from effector T cells. Activated and expanded Treg cells repress the immune response, thereby preventing effector T-cell overreaction [36, 37]. To exclude the feasibility of interference brought by the interaction between Treg and Tcon cells, bone marrow chimera model was employed, by which we demonstrated evidence that Ubc9 deficiency impacts CD4 T-cell proliferation in the peripheral in an intrinsic manner. We thus further generated Treg-specific Ubc9-knockout mouse model, and Treg cells derived from Treg-KO mice exhibited diminished suppressive function and age-dependent decline of Treg number, which were coupled with spontaneous autoimmunity.

Interestingly, a similar phenotype was observed in PDPK1-deficient mice. Loss of PDPK1 thwarts thymocytes at DN4 stage, without perceptible effect on DP and SP cell development. However, peripheral CD4 T cells are decreased, and Treg cells reduce in number upon PDPK1 conditional ablation [23, 24]. It is worthy of note that PDPK1 is not the only factor involved in SUMOylation-mediated regulation of thymocyte development. For instance, SENP1 is highly expressed in DN T cells, and SUMOylation of STAT5 blocks its acetylation and subsequent signaling critical for T cells going through DN4 stage [38]. Moreover, UBC9 mediates SUMOylation of NFAT1 to regulate thymocyte development at later DP stage [30, 39]. Collectively, these data including ours suggest that SUMOylation is a dynamic process in regulating CD4 T-cell biology. From early DN stage to later DP cells and peripheral matured cells, different protein substrates are responsible for the regulatory effect.

Another interesting discovery is that SUMOylation likely exerts a complex effect on distinct T-cell subsets. Mice with CD4 T-cell PDPK1 deficiency manifest inflammatory symptoms [23], while our Ubc9 KO mice are featured by lymphoid atrophy. Under both circumstances, Treg cells are comparably impaired, and therefore, the discrepancy is possibly due to the difference in Tcon cells. Indeed, CD4 emerged at DN4 stage, and PDPK1 does not affect later DP stage, other factors act in early DN stage (e.g., NFAT1), all of which lead to developmental problem that may explain the perceived discrepancy (Fig. S2B). In addition, we did not see any difference in terms of CD4 T cell lineage commitment, and transduction of SUMOylation-defective PDPK1 failed to affect CD4 T-cell differentiation as well. However, the implication of additional SUMOylation targets cannot be excluded [40–42]. For example, SUMOylation of BACH2 and RORγt contributes to Treg and Th17 program, respectively [43–45]. Therefore, additional studies would be necessary to unravel more specific details on how SUMOylation regulates distinct effector T-cell subsets.

## Materials and methods

### Mice

Ly5.1 (CD45.1) mice were obtained from the Jackson Laboratory (Bar Harbor, ME, USA). Congenic CD45.2 Ubc9^fl/fl^ mice were backcrossed with CD4^cre^ or Foxp3^cre-eGFP^ transgenic mice to generate CD4 T-cell or Treg-cell-specific Ubc9-knockout (CD4^cre^; Ubc9^fl/fl^, KO; Foxp3^cre-eGFP^; Ubc9^fl/fl^, Treg-KO) mice and control Ubc9^fl/fl^ littermates. All mice were bred in the Tongji Medical College Animal Center with a 12/12 h light/dark cycle (Wuhan, China) in a specific pathogen-free (SPF) facility. Since KO mice showed dramatic reduction of peripheral CD4 T cells with time, 4–5-week-old male mice were sacrificed for experimental purpose to get enough CD4 T cells. All experiments were approved by the Animal Care and Use Committee (ACUC) of Tongji Hospital and conducted in accordance with NIH guidelines. No randomization or blinding was used in animal studies.

### Antibodies and reagents

Recombinant murine IL-4 (#214-14), IL-12 (#210-12), IL-2 (#212-12), IL-23 (#200-23), IL-1β (#211-11B), and TGF-β (#100-21) were obtained from PeproTech (Rocky Hill, Connecticut, USA). Anti-CD3 (clone 145-2C11, #553057) and anti-CD28 (clone 37.51, #553295) were purchased from BD Bioscience (Franklin Lakes, NJ, USA). Anti-UBC9 (#4786), anti-PDPK1 (#5662), anti-p-PDPK1 (Ser241) (#3438), anti-SUMO1 (#4930), anti-AKT (#9272), anti-p-AKT (Ser473) (#4058), anti-p-AKT (Thr308) (#9275), anti-mTOR (#2983), anti-p-mTOR (Ser2448) (#2971), anti-p-p70S6K (#9234 S), and anti-β-Actin (#4970) antibodies were purchased from Cell Signaling Technology (Danvers, MA, USA). PE-conjugated anti-mouse CD45.1 (#110708), APC-conjugated anti-mouse CD45.2 (#109814), APC-conjugated anti-mouse IL-4 (#504106), FITC-conjugated anti-mouse CD4 (#100406), PE-conjugated anti-mouse CD8 (#100708), PerCP-conjugated anti-mouse CD8 (#100732), PerCP-conjugated anti-mouse CD44 (#103036), APC-conjugated anti-mouse CD62L (#104412), APC-conjugated anti-mouse IL-17A (#506916), PE-conjugated anti-mouse IL-17A (#506904), APC-conjugated anti-mouse IFN-γ (#505810), PE/Cy7-conjugated anti-mouse IFN-γ (#505826), PE-conjugated anti-mouse Ki67 (#151209), Brilliant Violet 421™ conjugated anti-mouse CD304 (Neuropilin-1) (#145209), Alexa Fluor® 647-conjugated anti-mouse Foxp3 (#126408), Anti–IFN-γ (XMG1.2), and anti–IL-4 (11B11) were obtained from Biolegend (San Diego, CA, USA).

### Flow cytometry analysis

Single-cell suspension was obtained from thymus or spleen and cell-surface markers were stained in 1 × PBS containing 2% BSA on ice with indicated antibodies. Intracellular staining was performed using the Transcription Factor Buffer Set (BD Biosciences, 562574) with indicated antibodies. For intracellular cytokine staining, cells were stimulated for 5 h with a mixture containing phorbol 12-myristate 13-acetate (PMA) (50 ng/mL, Sigma, St. Louis, USA) and ionomycin (1 μg/mL, Sigma), and then treated for another 1 h with Golgi-Plug (BD Biosciences). Indirect phospho-flow staining was performed following the manufacturers’ instructions (Biolegend: Fixation Buffer, 420801; True-Phos Perm Buffer, 425401). Data collection was performed with a MACS Quant Analyzer10 (Miltenyi Biotec, Germany), and data analysis was done with FlowJo software version 10 (Tree Star).

### In vitro T cell differentiation and proliferation assay

Naive T cells from WT control and KO mice were isolated using mouse naive CD4 T cell isolation kit (Miltenyi Biotec, 130-104-453). Cells were labeled with the tracing dye CFSE (C34554, Thermo Fisher, South San Francisco, CA, USA) according to the suggested protocol, and then subjected to plate bound anti-CD3 (10ug/ml) and anti-CD28 (5ug/ml) stimulation under various lineage commitment conditions: (Th1: IL-12 10 ng/ml + Anti-IL-4 10ug/ml; Th2: IL-4 20 ng/ml + Anti-IFN-γ 10ug/ml; Th17: IL-6 50 ng/ml + TGF-β 2 ng/ml + IL-1β 10 ng/ml + IL-23 10 ng/ml + Anti-IFN-γ 10ug/ml + Anti-IL-4 10ug/ml; Treg: IL-2 100 U/ml + TGF-β 5 ng/ml + Anti-IFN-γ 10ug/ml + Anti-IL-4 10ug/ml). Three to five days later, cells were harvested for flow cytometry analysis.

### Generation of bone marrow chimera

For bone marrow (BM) transplantation experiment, recipient CD45.1 (WT) mice were lethally irradiated (1000–1100 cGy, two split doses, 4 h apart) and each mouse was intravenously injected 1 × 10^7^ total mixed BM cells from CD45.1 (WT) and CD45.2 (KO) mice at the ratio of 1:1. Nutritional gel packs were provided in each cage and antibiotics (Gentamicin) in the drinking water for the duration of the experiment. The mice were sacrificed for further analysis eight weeks after the transplantation.

### Seahorse metabolic analysis and glucose uptake assay

For Seahorse assay, plates were coated with poly-D-lysine at 4°C overnight and 1 × 10^6^ CD4 T cells or plasmid-transfected HEK293T cells were seeded per well, followed by brief centrifugation (450 g, 30 s) to make cells evenly adhere to the plate. OCR was measured on a Seahorse XF96 analyzer (Agilent, Santa Clara, CA, USA) in the presence of the mitochondrial inhibitor Oligomycin (1 μM), mitochondrial uncoupler FCCP (1 μM), and respiratory-chain inhibitor Antimycin A/Rotenone (2 μM). ECAR was measured in the presence of glucose (10 mM), Oligomycin (1 μM), and 2-DG (50 mM). For glucose-uptake assay, PBMC or CD4 T cells were isolated and starved in glucose-free medium for 45 min. 2-NBDG was incorporated into the culture medium at a final concentration of 100uM for 1 h and then subjected for flow-cytometry analysis.

### Real-time PCR and Western blot analysis

Total RNA was extracted from cells using the Trizol reagent (Takara, Japan) following the manufacturer’s instructions. cDNA was obtained with a reverse transcription kit (Applied Biosystems, Foster City, CA, USA). The expression of Glut1, Eno1, Pkm2, and Pgk1 was analyzed by qPCR using the SYBR Green PCR Master Mix (Applied Biosystems) and normalized to the expression of β-Actin. The following primers were used: β-Actin: 5′-CAT TGC TGA CAG GAT GCA GAA GG -3′, reverse: 5′-TGC TGG AAG GTG GAC AGT GAG G -3′; Glut1 forward: 5′-GCT TCT CCA ACT GGA CCT CAA AC -3′, reverse: 5′-ACG AGG AGC ACC GTG AAG ATG A -3′; Eno1 forward: 5′-TAC CGC CAC ATT GCT GAC TTG G -3′, reverse: 5′-GCT TGT TGC CAG CAT GAG AAC C -3′; Pkm2 forward: 5′-CAG AGA AGG TCT TCC TGG CTC A -3′, reverse: 5′-GCC ACA TCA CTG CCT TCA GCA C -3′; Pgk1 forward: 5′- GAT GCT TTC CGA GCC TCA CTG T -3′, reverse: 5′-ACC AGC CTT CTG TGG CAG ATT C -3′. Total proteins were prepared from cells using RIPA lysis buffer (Beyotime, Shanghai, China) containing protease inhibitors (Roche, IN, USA). Western blot analysis was carried out as reported by probing the blots with indicated primary antibodies followed by an HRP-conjugated secondary antibody. The reactive bands were visualized using an ECL Plus TM Western blot kit. β-Actin was used for normalization.

### Plasmid constructs and transfection

Expression plasmids for full-length human PDPK1 (also known as PDK1), UBC9 and SUMO1 were generated using standard cloning procedures. PDPK1-MU were generated using site-directed mutagenesis kit (Takara, Japan) and confirmed by pyro-sequencing. Human embryonic kidney (HEK)293T cells were transferred from the Center for Biotechnology and Genomic Medicine, Georgia Regents University (now named Augusta University, Augusta, GA, USA). Cells were transfected with the above-prepared plasmid using the Lipofectamine 3000 reagent (Invitrogen, Irvine, CA, USA), and harvested for analysis 48 h after transfection.

### PDPK1 SUMOylation analysis

HEK293T cells (ATCC, free for mycoplasma and authenticated by STR profiling) were transfected with either PDPK1-WT or PDPK1-MU plasmid. After washes in ice-cold phosphate-buffered saline (PBS), the cells were lysed on ice for 30 min in an IP lysis buffer (50 mM Tris-HCl, pH 7.5, 150 mM NaCl, 1% NP-40, 5 mM EDTA, and 0.1% SDS) containing protease inhibitors (10 μg/ml aprotinin, 10 μg/ml leupeptin and 1 mM PMSF), phosphatase inhibitors (5 mM sodium pyrophosphate and 1 mM Na3VO4), and 20 mM N-ethylmaleimide (Sigma, St Louis, MO, USA). The cell lysates were precleared with protein-G agarose beads (GE Healthcare, New York, USA) for 1 h and then incubated with 5 µg of anti-SUMO1 or anti-PDPK1 antibody overnight, and proteins were then immunoprecipitated for an additional 4 h at 4 °C with protein-G beads. The samples were probed with the indicated antibodies (anti-SUMO1 or anti-PDPK1) for immunoblotting analysis.

### Phospho-proteomic study of PDPK1 activity

Lysis buffer containing 8 M urea, 1% protease, and phosphatase-inhibitor cocktail was added into each sample, which was then sonicated three times on ice using a high intensity ultrasonic processor. The remaining cell debris was removed by centrifugation at 12,000 g at 4°C for 10 min. Finally, the supernatant was collected and the protein concentration was determined with BCA kit according to the manufacturer’s instructions.

For trypsin digestion, the protein solution was reduced with 5 mM dithiothreitol for 30 min at 56°C and alkylated with 11 mM iodoacetamide for 15 min at room temperature in darkness. The protein sample was then diluted by adding 100 mM TEAB to urea concentration less than 2 M. Finally, trypsin was added at 1:50 trypsin-to-protein mass ratio for the first digestion overnight and 1:100 trypsin-to-protein mass ratio for the second 4 h digestion.

After digestion, peptide was desalted by Strata X C18 SPE column and vacuum-dried. Peptides were reconstituted in 0.5 M TEAB and processed according to the manufacturer’s protocol for TMT kit. After TMT labeling, peptides were fractionated into 60 fractions by high-pH reverse-phase HPLC, and then combined into 9 fractions and dried by vacuum centrifuging.

To enrich phosphate-modified peptides, tryptic peptides dissolved in enrichment buffer (50% acetonitrile and 6% trifluoroacetic acid) were incubated with pre-washed IMAC microspheres at 4°C overnight with gentle shaking. After removing nonspecifically absorbed peptides through washing, elution buffer containing 10% NH4OH was added. The supernatant containing phosphopeptides was collected and lyophilized for LC–MS/MS analysis, which operated on EASY-nLC 1000 UPLC system and in Q Exactive TM Plus (Thermo). The resulting MS/MS data were processed using Maxquant search engine (v.1.5.2.8). Finally, KEGG pathway analysis and functional enrichment were conducted to further interpret the data.

### Virus transduction of CD4 T cells

Naive T cells or Treg cells were isolated from spleen of WT mice with mouse naive CD4 T cell isolation kit (Miltenyi Biotec, 130-104-453) or mouse CD4 CD25^+^ regulatory T cell isolation kit (Miltenyi Biotec, 130-091-041) according to the manufacturer’s instructions. The lentiviruses carrying human PDPK1 gene (PDPK1-WT) or SUMOylation mutant (PDPK1-MU) were packaged by Han Biotech Co., Ltd. (Shanghai, China) as previously reported [46]. Briefly, CD4 T cells were pre-activated with plate-bound anti-CD3 (10ug/ml) and anti-CD28 (5ug/ml) for 24 h, then spin-transduced with corresponding viruses (30°C, 450 g x 90 min) in the presence of 5ug/ml polybrene. Cells were then cultured for another 6 h in the incubator before fresh medium was changed. Second-round transduction was conducted similarly, except for shortening the incubation time from 6 h to 2 h. For proliferation assay, cells were rested for 24 h, and then reseeded for 48 h plate-bound anti-CD3 + anti-CD28 stimulation. Intracellular Ki67 staining was conducted to determine the proliferative population within the GFP^+^ gate. For differentiation assay, lineage-commitment cocktails were added from the very beginning. After virus transduction, cells were cultured in fresh medium supplemented with lineage-commitment cocktails (without TCR stimulation) for another 2 days. Intracellular staining was applied to determine the differentiation efficiency within the GFP^+^ gate.

### Statistical analysis

The sample sizes were empirically determined by consulting relevant studies (including animal studies). Data were expressed as mean ± SD. All statistical analyses were carried out using the Graphpad Prism 5.0 software (La Jolla, CA, USA). The data were analyzed by Student’s *t*-test or one-way or two-way ANOVA where indicated. In all cases, *p* < 0.05 was considered as statistically significant.

## Supplementary information

Supplementary Figure Legends

Reproduction Checklist

Supplementary Figure 1

Supplementary Figure 2

Supplementary Figure 3

Supplementary Figure 4

Supplementary Figure 5

Supplementary Table

Supplemental material for WB

## Data Availability

All data needed to evaluate the conclusions in this article are included in the paper and/or its supplementary information (raw bands for Western blot were included). Additional data related to this paper may be requested from the authors.
