# Supplementary material for: SUMOylation of PDPK1 Is required to maintain glycolysis-dependent CD4 T-cell homeostasis
Source: Cell Death Dis. 2022 Feb 24;13(2):181. doi: 10.1038/s41419-022-04622-1 (PMC8873481; doi:10.1038/s41419-022-04622-1)
Supplement: Supplementary file 1 — Supplementary Figure Legends [file 41419_2022_4622_MOESM1_ESM.docx]

**Supplementary Figure Legends**

**Figure S1**: Ubc9 deficiency impairs CD4 T cell proliferation. (A-B) Detection of thymus DN stages was carried out by CD44 and CD25 staining. Percentage of each subset (DN1: CD44^+^CD25^-^; DN2: CD44^+^CD25^+^; DN3: CD44^-^CD25^+^; DN4: CD44^-^CD25^-^) was shown (n=3). (C) Apoptosis of thymus DP (WT: 11.51 ± 0.89% vs. KO: 12.00 ± 1.55%, *p* = 0.79) and CD4 SP cells (WT: 12.93 ± 0.53% vs. KO: 5.08 ± 0.39%, *p* < 0.001) was determined by Annexin V staining (n=4). (D) Foxp3^+^ Treg cells were further divided into pTreg and tTreg by Nrp1 staining (n=3). nTreg/pTreg in WT mice: 68.88 ± 1.31%/28.43 ± 2.19% vs. 31.13 ± 1.31%/59.53 ± 1.76% in KO mice. The p-value was determined by Student’s unpaired t test.

**Figure S2**: Ubc9 deficiency leads to lymphoid organ atrophy. (A) Absolute number of CD4 and CD8 T cells in WT and KO spleen. (B) Absolute number of CD4 T cells in WT and KO bone marrow. (C-F) Absolute cell count of Th1, Th2, Th17, Treg subsets in WT and KO spleen. For all graphs, the mean ± SD is shown from 3 mice. The p-value was determined by Student’s unpaired t test.

**Figure S3**: Ubc9 deficiency impairs PDPK1 signaling. (A) Phosphorylation level of S6K in WT and Ubc9 deficient CD4 T cells (MFI: WT: 2,293 ± 110.6 vs. KO: 1,260 ± 58.74, *p* < 0.001) (n=4). (B) Phosphorylation level of PDPK1 in WT and Ubc9 deficient Treg cells (MFI: WT: 3,023 ± 160.9 vs. KO: 1,853 ±41.10, *p* < 0.001) (n=4). (C) Phosphorylation level of S6K in WT and Ubc9 deficient Treg cells (MFI: WT: 2,935 ± 112.4 vs. KO: 1,960 ± 83.17, *p* < 0.001) (n=4). The p-value was determined by Student’s unpaired t test.

**Figure S4**: PDPK1 activity is modulated by SUMOylation process. Gene ontology and motif analysis of differentially (MU/WT) phosphorylated proteins (A) Biological processes. (B) Molecular function. (C) Domains in up-regulated proteins (MU/WT). (D) Domains in down-regulated proteins (MU/WT).

**Figure S5**: SUMOylation of PDPK1 regulates glycolytic metabolism. (A) ECAR of WT PDPK1, MU PDPK1 and Vector transfected HEK293T cells at basal levels, followed by sequential treatment (dashed lines) of glucose (Glc), Oligo and 2-DG. (B-D) Accordingly, baseline glycolysis (WT: 32.00 ± 2.61mpH/min vs. MU: 20.73 ± 1.55mpH/min, *p* < 0.01), maximal glycolytic capacity (WT: 246.7 ± 21.51mpH/min vs. MU: 130.5 ± 12.14mpH/min, *p* < 0.01) and glycolytic reserve (WT: 214.6 ± 18.93mpH/min vs. MU: 109.8 ± 12.05mpH/min, *p* < 0.01) were shown (n=5, representative of two experiments).
