# Supplementary figures and images for: SUMOylation of PDPK1 Is required to maintain glycolysis-dependent CD4 T-cell homeostasis
Source: Cell Death Dis. 2022 Feb 24;13(2):181. doi: 10.1038/s41419-022-04622-1 (PMC8873481; doi:10.1038/s41419-022-04622-1)

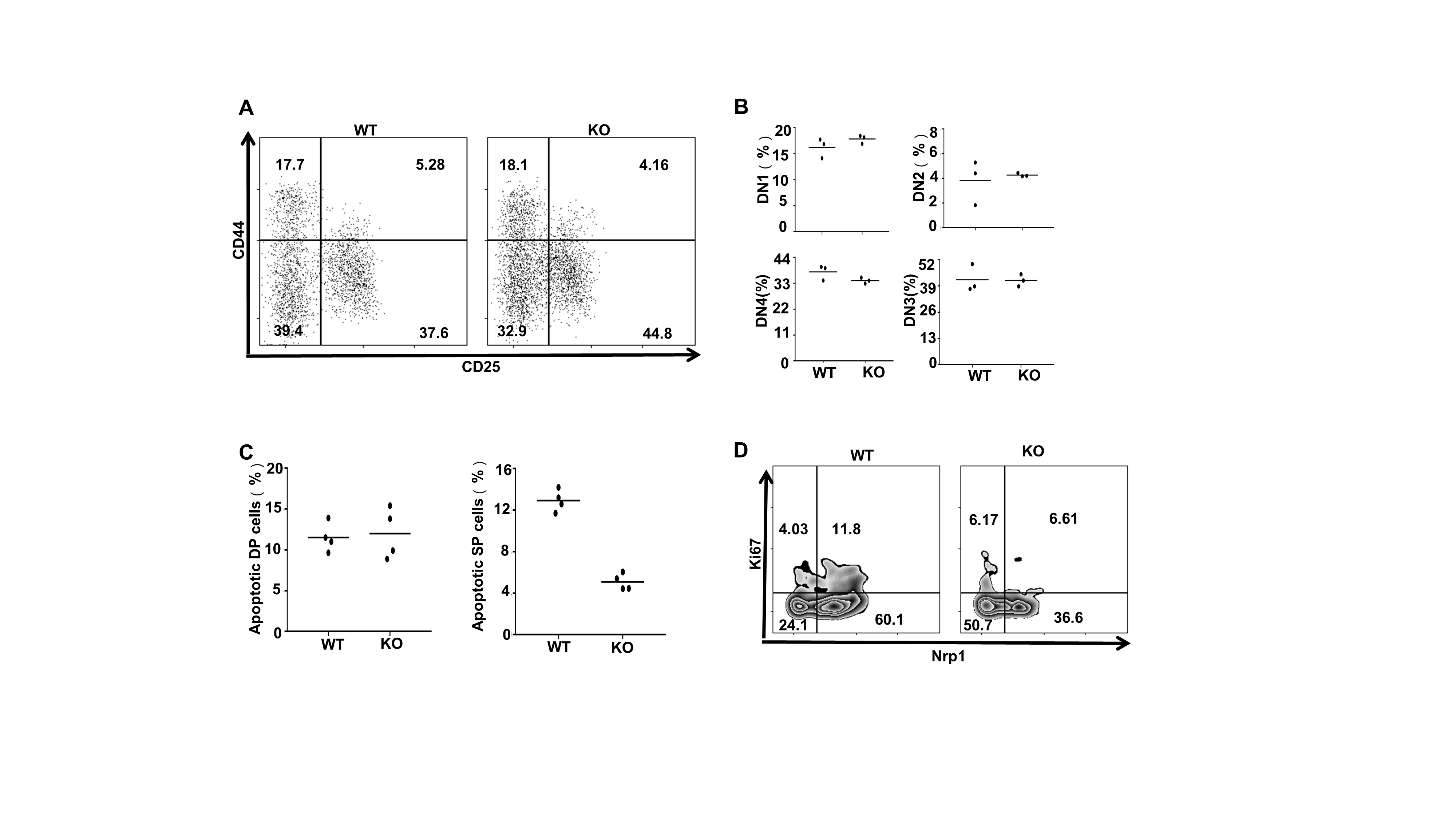

Supplement: Supplementary file 3 — Supplementary Figure 1 [file 41419_2022_4622_MOESM3_ESM.tif]

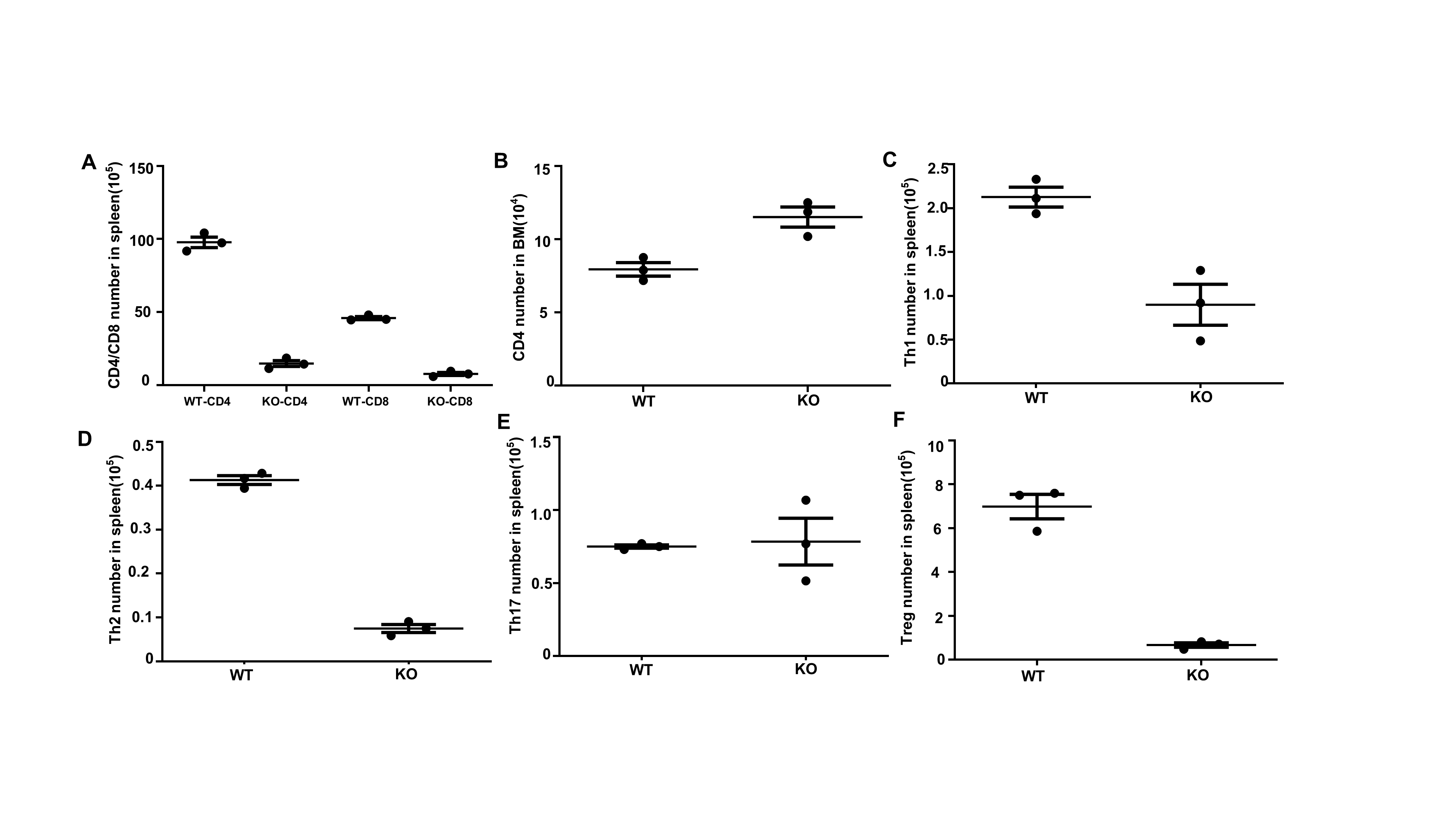

Supplement: Supplementary file 4 — Supplementary Figure 2 [file 41419_2022_4622_MOESM4_ESM.tif]

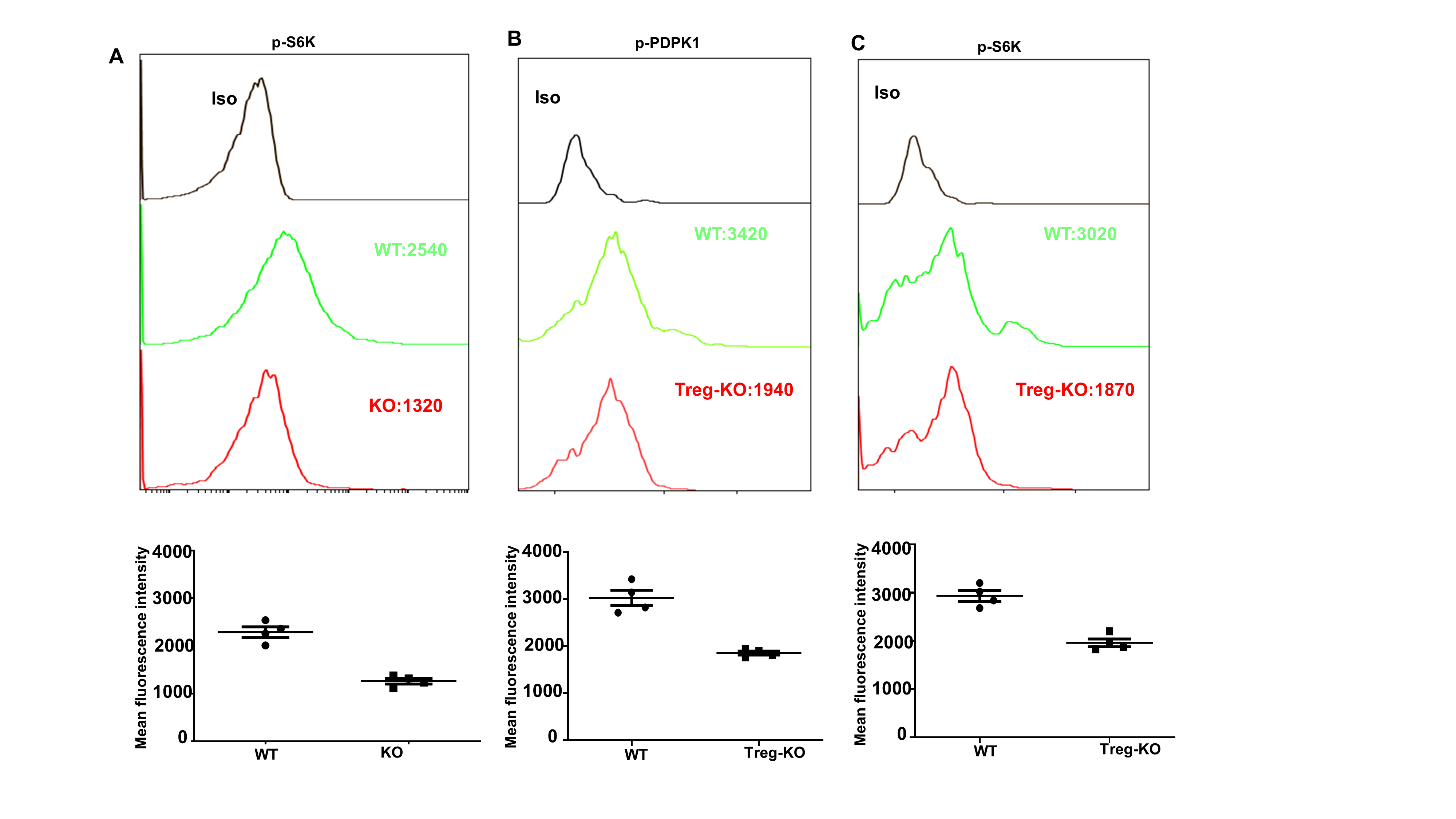

Supplement: Supplementary file 5 — Supplementary Figure 3 [file 41419_2022_4622_MOESM5_ESM.tif]

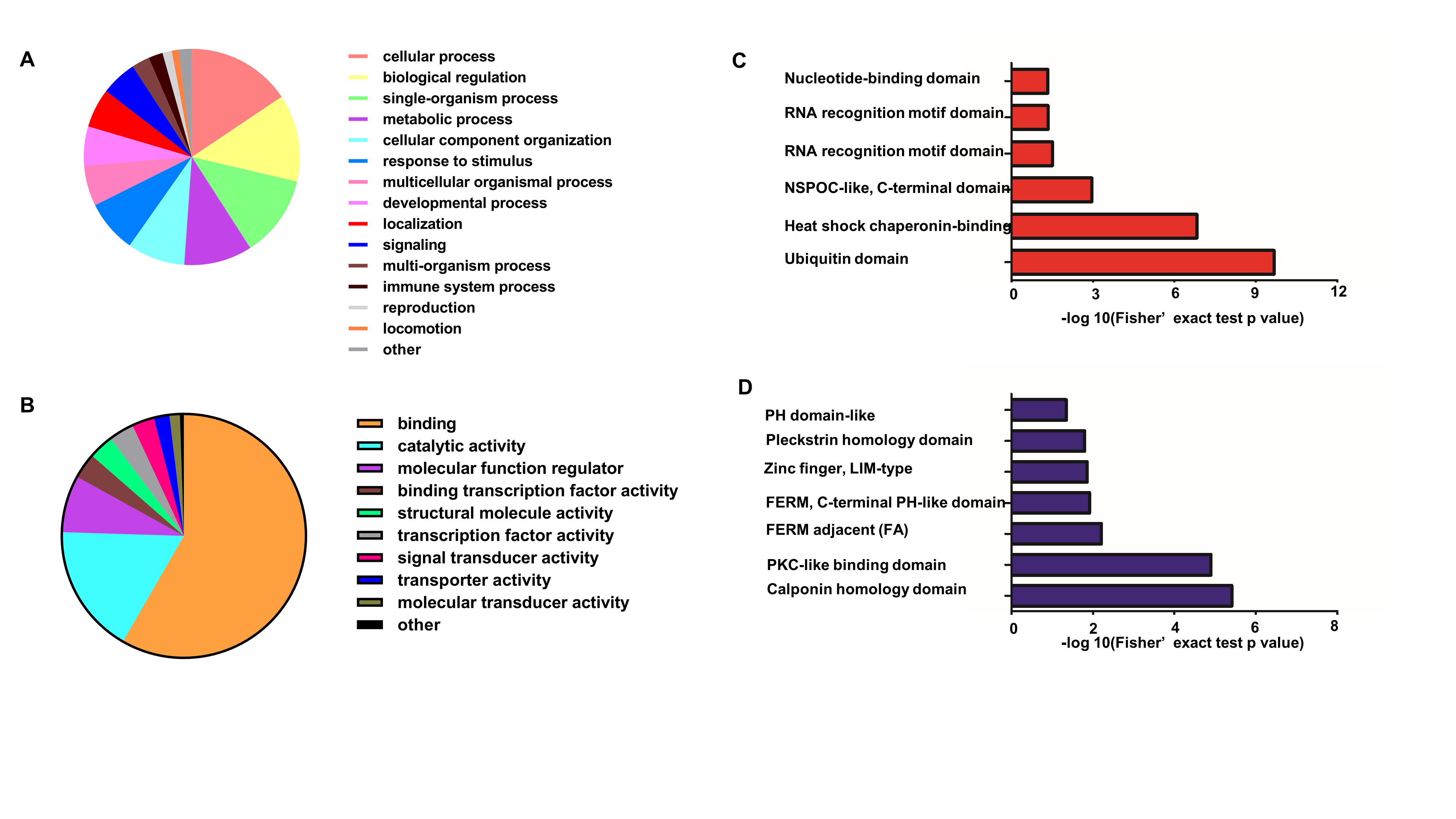

Supplement: Supplementary file 6 — Supplementary Figure 4 [file 41419_2022_4622_MOESM6_ESM.tif]

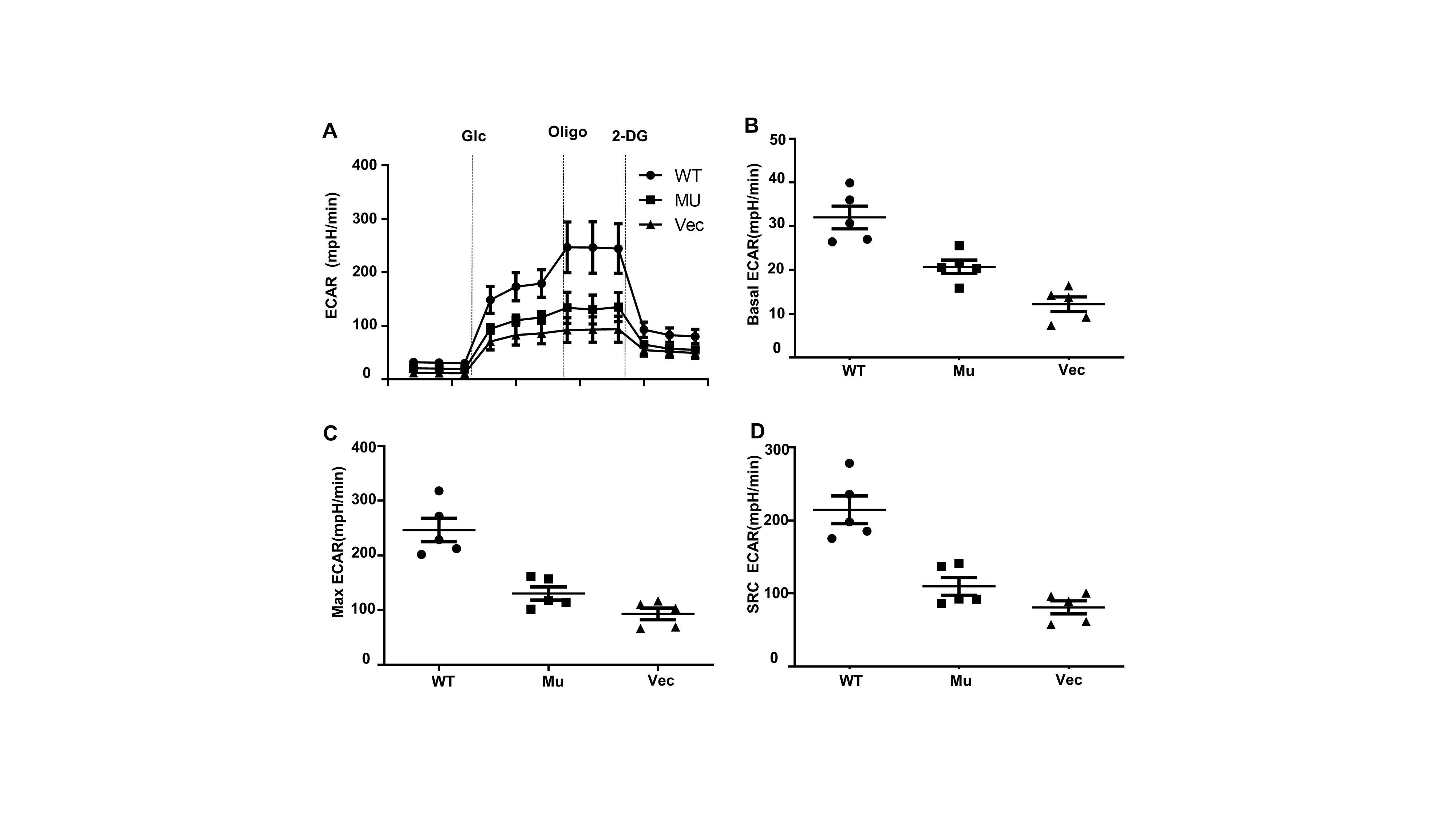

Supplement: Supplementary file 7 — Supplementary Figure 5 [file 41419_2022_4622_MOESM7_ESM.tif]
