# Supplementary material for: SUMOylation of PDPK1 Is required to maintain glycolysis-dependent CD4 T-cell homeostasis
Source: Cell Death Dis. 2022 Feb 24;13(2):181. doi: 10.1038/s41419-022-04622-1 (PMC8873481; doi:10.1038/s41419-022-04622-1)

Figure 5A, Western Blot Bands :

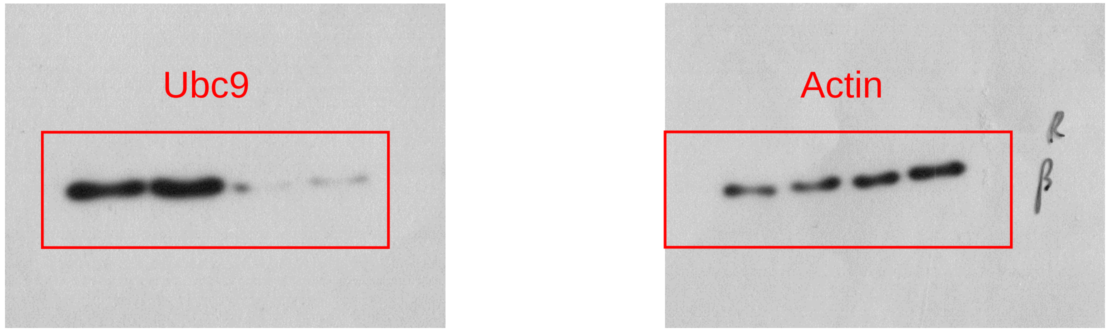

Figure 5B, Western Blot Bands :

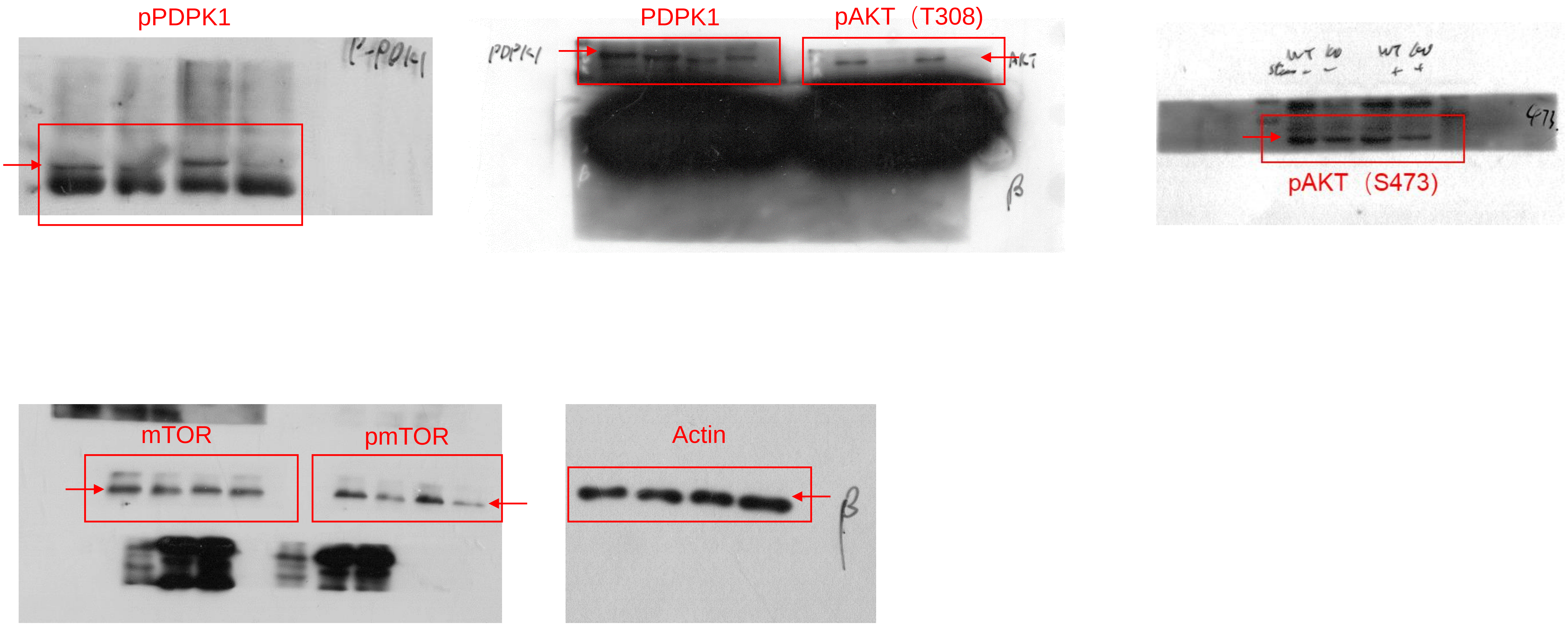

Figure 7A, Western Blot Bands :

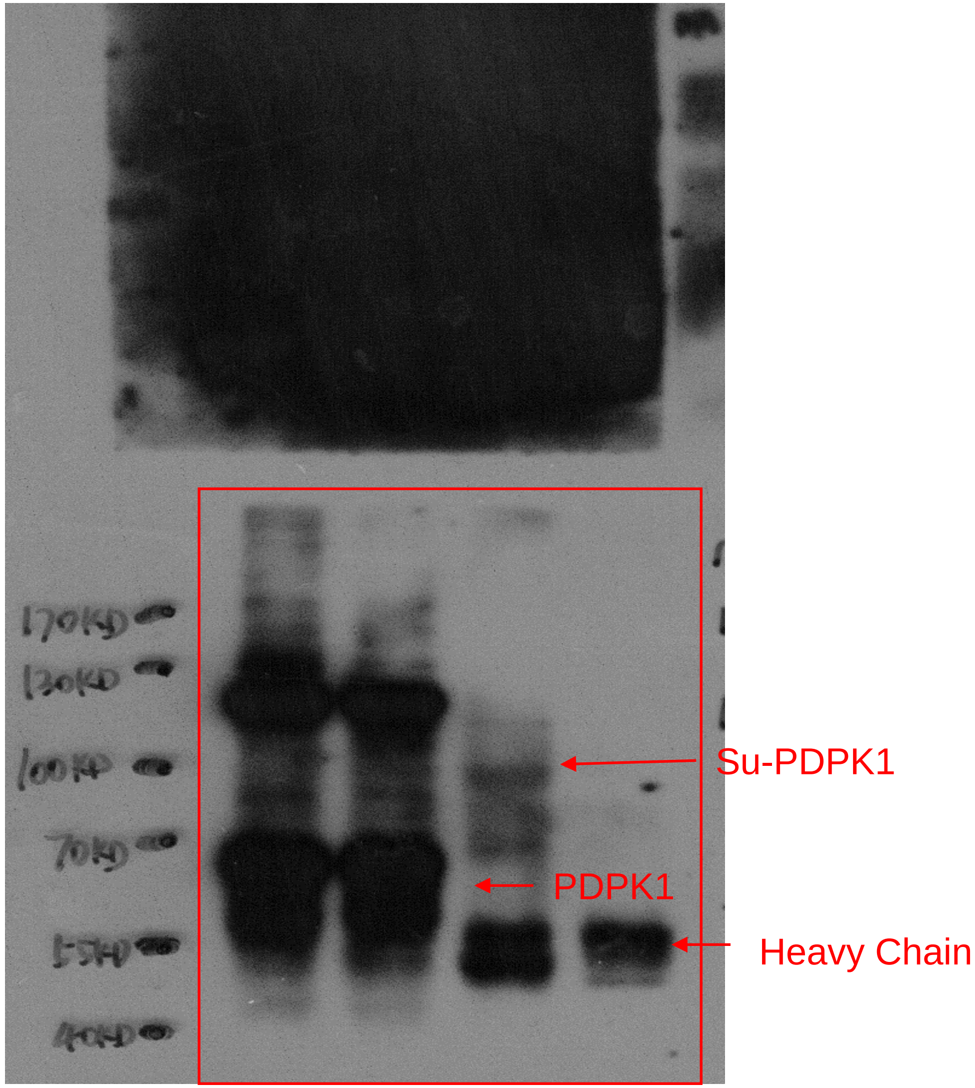

Figure 7C, Western Blot Bands :

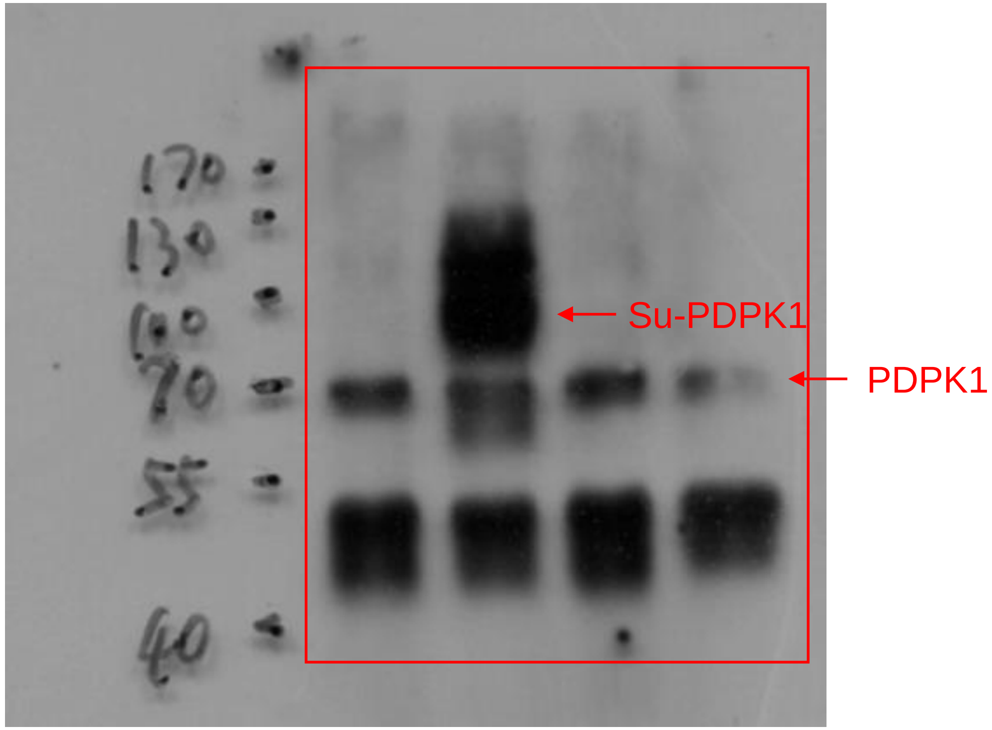

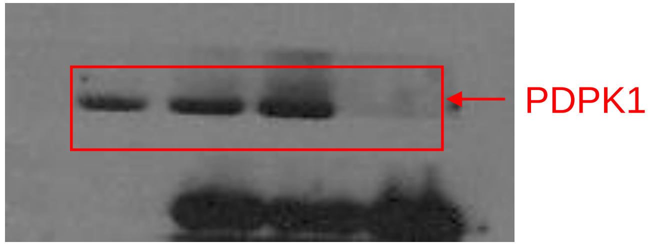

Figure 7D, Western Blot Bands :

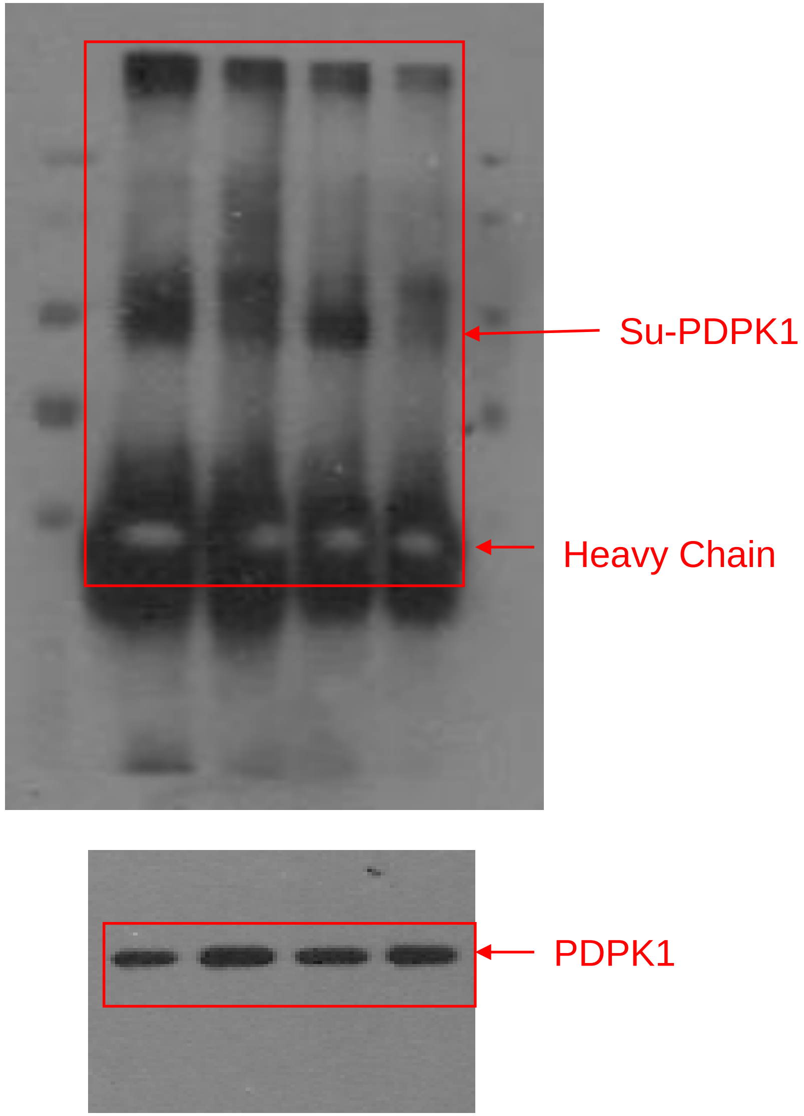

Figure 7E, Western Blot Bands :

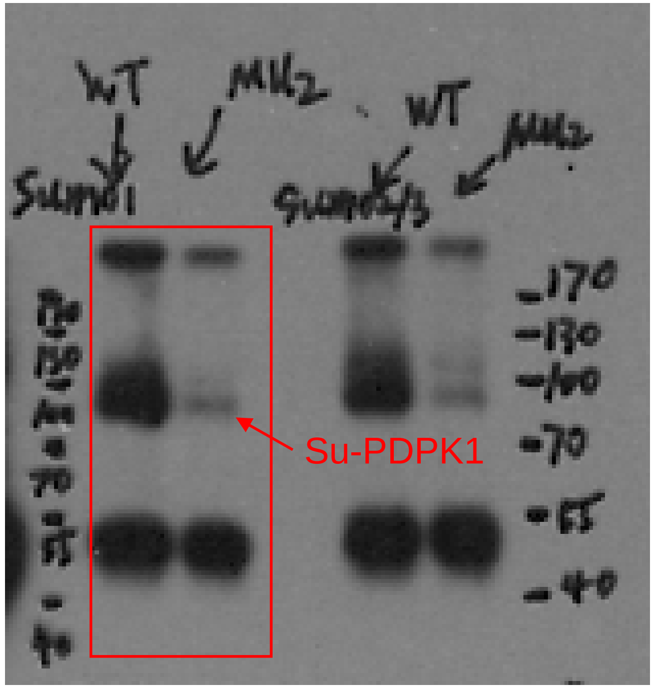

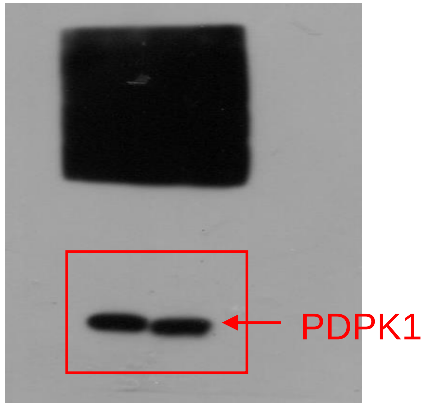

Figure 7A, Western Blot Bands :

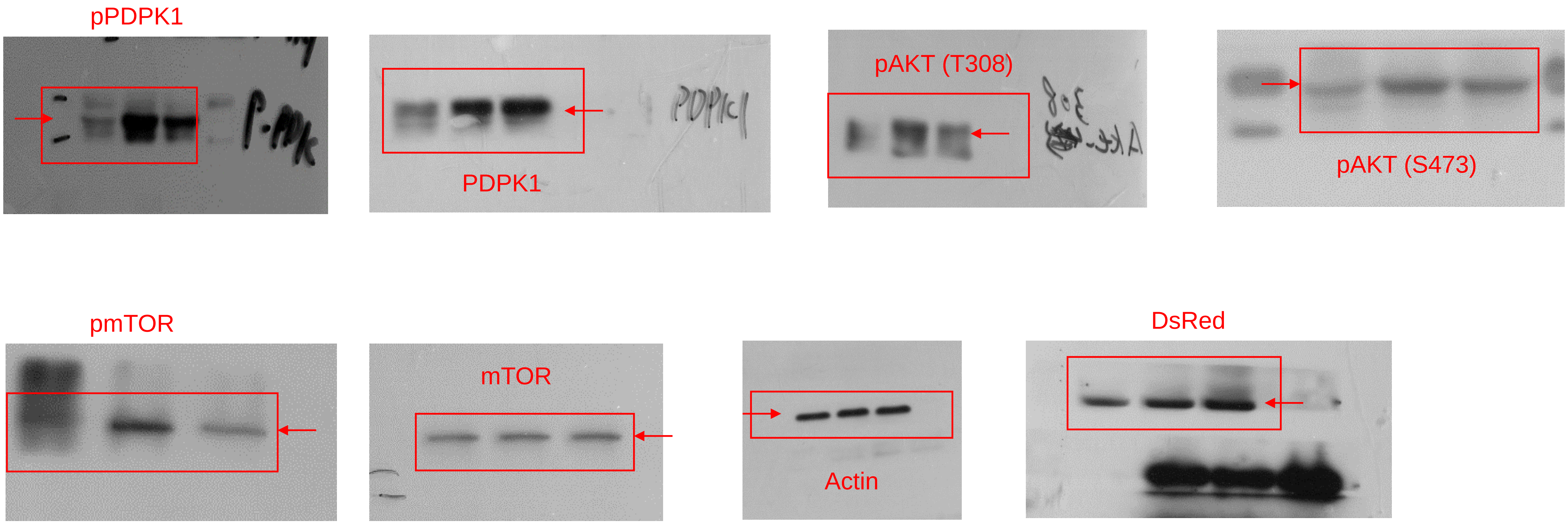

Supplement: Supplementary file 9 — Supplemental material for WB [file 41419_2022_4622_MOESM9_ESM.pdf]
